# A Data‐Driven Closed‐Loop Control Approach to Drive Neural State Transitions for Mechanistic Insight

**DOI:** 10.1002/hbm.70600

**Published:** 2026-07-15

**Authors:** Niklas Emonds, Evelyn Herberg, Martin Fungisai Gerchen, Marc Pritsch, Joshua Rocha, Vera Zamoscik, Peter Kirsch, Roland Herzog, Georgia Koppe

**Affiliations:** ^1^ Hector Institute for AI in Psychiatry & Department of Psychiatry and Psychotherapy, Central Institute of Mental Health (CIMH), Medical Faculty Mannheim Heidelberg University Heidelberg Germany; ^2^ Interdisciplinary Center for Scientific Computing Heidelberg University Heidelberg Germany; ^3^ Department of Clinical Psychology, CIMH, Medical Faculty Mannheim Heidelberg University Mannheim Germany; ^4^ Department of Psychology University of Heidelberg Heidelberg Germany; ^5^ German Center for Mental Health, DZPG Partner Site Mannheim‐Heidelberg‐Ulm Mannheim Germany; ^6^ Department of Psychology, School of Social Sciences University of Mannheim Mannheim Germany; ^7^ Hertie Institute for AI in Brain Health University of Tübingen Tübingen Germany

**Keywords:** affective state transitions, brain network controllability, closed‐loop neuromodulation, dynamical systems reconstruction, major depressive disorder, optimal control

## Abstract

Altered affective state dynamics are a characteristic feature of depression and can persist beyond symptomatic remission. Individuals with remitted major depressive disorder (rMDD) often show heightened reactivity to negative mood states and reduced efficiency in recovering from them, consistent with changes in affective dynamics after remission. These patterns may reflect alterations in the brain's capacity to flexibly shift between neural states that support distinct affective modes. Characterizing the dynamical mechanisms that govern transitions into and out of experimentally induced affective states is therefore essential for understanding vulnerability to recurrence and informing mechanistic interventions. We developed a data‐driven framework combining dynamical system reconstruction (DSR) with model‐based control to infer optimal control policies for transitions between resting and sad mood brain states using functional magnetic resonance imaging (fMRI) data. Nonlinear DSR models trained on individuals with rMDD and healthy controls (HC) yielded region‐specific, state‐dependent control strategies. Small regions (e.g., sgACC, NAcc) showed higher controllability, requiring less energy for state transitions. Notably, rMDD participants required less control energy than HC to shift both into and, to a more spatially restricted extent, out of sad mood states. Despite reaching the resting state target with similar proximity, however, they remained closer to the sad mood distribution when returning to rest, reflecting a residual bias toward the sad mood distribution. Elevated coupling in rMDD, especially toward the DLPFC, was linked to lower control energy, suggesting that stronger network coupling facilitates transitions. These findings indicate rMDD dynamics that ease entry into sad mood states but impede full disengagement. More broadly, they demonstrate how closed‐loop control applied to data‐driven dynamical models can provide mechanistic insight into brain state transitions and inform future hypotheses about cognitive vulnerability or compensatory processes.

## Graphical abstract

The study introduces a data‐driven framework combining dynamical systems reconstruction and closed‐loop control to analyze brain‐state transitions in remitted depression. Using fMRI data, we show that these individuals more easily enter but struggle to exit sad mood states, revealing altered connectivity and potential neuromodulation targets for depression treatment.

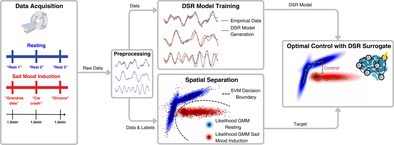

## 1 Introduction

Altered affective state dynamics are a central feature of major depressive disorder (MDD). Even after symptomatic remission, individuals with a history of MDD frequently exhibit heightened sensitivity to negative mood states, persistent affective bias, and difficulties disengaging from induced sadness (Spinhoven et al. 2018; Timm et al. 2017). A large body of neuroimaging work has demonstrated enduring alterations in emotion‐related and default mode networks in MDD, suggesting that vulnerability to depression may be reflected in network‐level reorganization that persists beyond acute episodes (Demirtaş et al. 2016; Gou et al. 2023; Kaiser et al. 2015; Schmaal et al. 2016). However, most such evidence relies on static or time‐averaged measures of connectivity, which provide limited insight into how brain activity unfolds over time. A mechanistic understanding of the temporal evolution of affective brain states therefore remains an open challenge.

Previous studies have associated depressive vulnerability, negative affect (NA), and maladaptive self‐referential processing with altered functional connectivity across large‐scale brain networks, particularly increased coupling between default mode and fronto‐parietal regions and decreased coupling with the salience network (Lydon‐Staley et al. 2019; Zamoscik et al. 2018; Zhu et al. 2012). Elevated levels of connectivity involving the parahippocampal gyrus (PHG), anterior cingulate cortex (ACC), and posterior cingulate cortex (PCC) have been especially prominent in remitted depression (Zamoscik et al. 2014). While these descriptive findings highlight network‐level alterations, they do not directly reveal the causal mechanisms that enable entry into and disengagement from negative affective states.

To uncover such mechanisms, generative models that simulate brain dynamics—and allow for controlled perturbation—have emerged as a powerful framework (Durstewitz et al. 2021, 2023; Luo et al. 2025). Within this framework, optimal control theory (Brunton and Kutz 2019) enables inference of the minimal interventions required to shift brain states under physiological constraints. Prior work has applied linear control models to empirical connectivity data to study cognitive transitions, predict symptom severity, or evaluate controllability in neurological conditions (Betzel et al. 2016; Deng et al. 2022; Lynn and Bassett 2019; Medaglia et al. 2017; Parkes et al. 2024; Yao et al. 2025; Zhou et al. 2025). Others have introduced biophysical or neural field models to simulate state transitions in conditions such as epilepsy (Costa et al. 2024; Deco et al. 2019; Salfenmoser and Obermayer 2022; Taylor et al. 2015). In mental disorder treatments, interventions have also been conceptualized as model predictive control problems (Brar et al. 2018; Chang et al. 2020; Santaniello et al. 2011; Steffen et al. 2024). While valuable, many of these approaches rely on simplified, often linear dynamics, which fail to capture phenomena such as multistability or chaos that characterize real neural systems (Breakspear 2017; Driscoll et al. 2024; Strogatz 2015; Volkmann et al. 2024), and therefore limit mechanistic interpretations. Models with greater biological detail often rely on handcrafted equations (Martínez et al. 2023; Taylor et al. 2015), which are typically not learned from data and are thus difficult to adapt or generalize across individuals and datasets.

Recent advances in dynamical systems reconstruction (DSR) offer a promising alternative. Data‐driven methods learn expressive, nonlinear models directly from time series data and can act as computational surrogates of neural systems (Brenner et al. 2025; Brunton and Kutz 2019; Durstewitz et al. 2023; Hess et al. 2023; Pathak et al. 2018). When combined with optimal control, such models make it possible to ask how reconstructed neural dynamics can be steered between empirically observed state distributions, and how much control energy—that is, how much modeled input—is required for these transitions.

Here, we leveraged this combination to investigate transitions between resting and induced sad mood states in remitted MDD (rMDD). Sad mood induction paradigms have been linked to sustained NA and increased rumination in remitted depression (Huffziger et al. 2013; Lydon‐Staley et al. 2019), yet the dynamical mechanisms that enable transitions into and out of these states remain poorly understood. Using fMRI data from participants with rMDD and matched healthy controls (HC) during a sad mood induction task, we trained DSR models that capture individual neural dynamics (Brenner et al. 2025; Volkmann et al. 2024). We then derived and analyzed optimal closed‐loop control policies within these reconstructed dynamics to characterize transitions between rest and sad mood in terms of the control energy required to steer the system from one empirical state distribution to another.

Specifically, our contributions are as follows. We present a novel data‐driven framework for inferring closed‐loop optimal control policies based on DSR models. We use model‐based simulations to examine control trajectories between resting and sad mood brain states, targeting single brain regions. Applying this framework to the present dataset, we found that inferred control policies differed between rMDD and HC individuals, reflecting altered neural dynamics. We identify key brain regions with elevated controllability and link these findings to underlying connectivity. In addition, we examine associations between individual differences in control energy and rumination to explore links between neural controllability and cognitive vulnerability. Together, these results provide a mechanistic characterization of altered affective state controllability in remitted depression and establish a foundation for future work linking neural dynamics to cognitive vulnerability processes such as repetitive negative thinking.

## 2 Results

**FIGURE 1:**
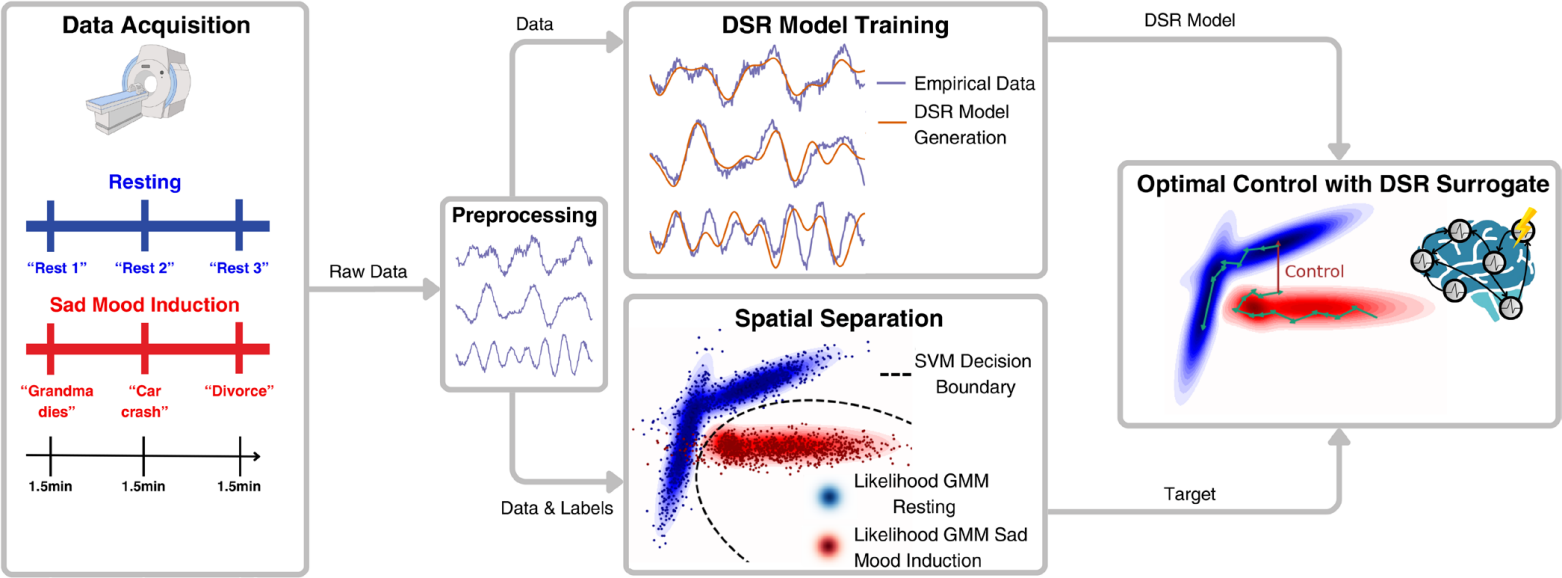
Neural activity was measured during two cognitive states, resting, and a sad mood induction (left, Zamoscik et al. 2014). After preprocessing, we proceeded with two parallel analyses: First, we trained DSR models on the empirical data to reconstruct the observed dynamics. Once trained, these models served as computational surrogates of the empirical dynamics and were used to generate simulated neural trajectories for the control analyses (center, top). Second, we treated the measured states as static data points to distinguish between resting and mood‐induction conditions (center, bottom). For this purpose, we trained Gaussian Mixture Models (GMMs) separately for each condition, obtaining probability distributions over the state space for both resting and mood‐induction states. These GMMs defined the control targets by assigning likelihoods to generated trajectories according to their proximity to resting or mood‐induction states. The loss function was defined as the negative log‐likelihood under the desired GMM. Control strategies are then learned by generating trajectories from the DSR model while systematically perturbing selected brain regions (right).

This study sought to derive optimal control policies from fMRI recordings collected from rMDD and HC individuals to gain mechanistic insights into the neural processes underlying transitions between resting and sad mood induction states. Figure 1 depicts the general procedure.

### 2.1 Spatial Separation Between Resting and Sad Mood Induction

**FIGURE 2:**
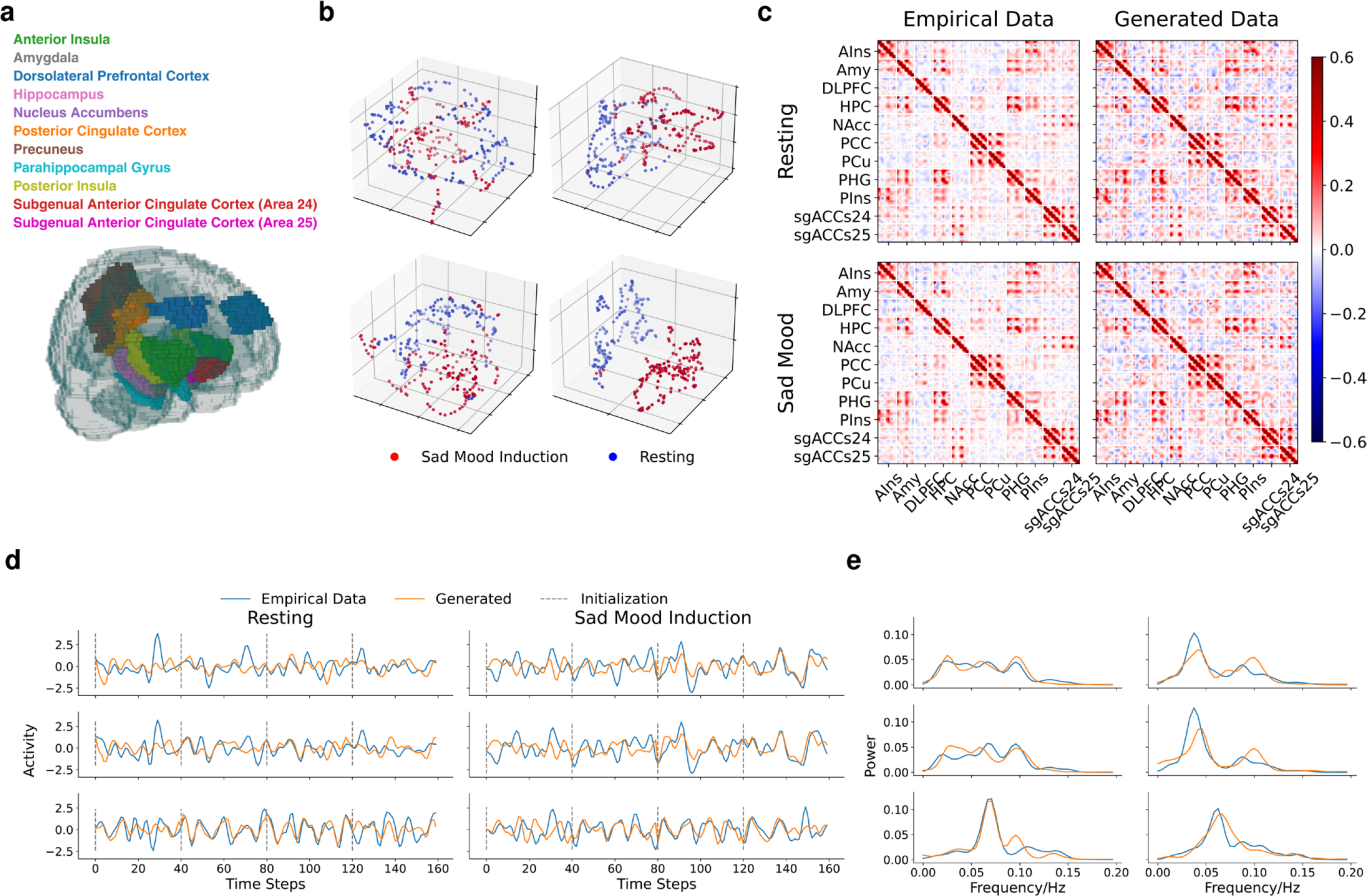
Neural state classifier and DSR model performance. (a) Eleven bilateral regions of interest (ROIs) implicated in depression, as visualized here, were selected for analysis. (b) Neural state representations projected via UMAP (McInnes et al. 2018), color‐coded by condition (resting: blue; sad mood induction: red) for selected participants. (c) Median cross‐correlation matrices between selected ROIs for empirical test data (left), and simulated test data from the DSR models (right). The observed connectivity patterns are consistent across empirical and simulated data. (d) Example time series of empirical neural activity (blue) and model reconstructions (orange) for a few representative time series. Gray dashed lines indicate the initialization period for the model‐generated trajectories. The final 40 time steps (test set) were not used during training. (e) Normalized power spectra of the time series shown in (d).

To characterize differences in the representation and distribution of the two cognitive states—required for downstream control analyses—we assessed separability between resting and sad mood induction states in neural state space using generative and discriminative classifiers (see Figure 2a and Section 5.2.1 for details on the preselected regions of interest; ROIs). Performance was evaluated under two complementary cross‐validation schemes: shuffled five‐fold cross‐validation with 25% held‐out test data to assess separability in state space, and temporally structured five‐fold cross‐validation using contiguous time segments to provide a conservative estimate that preserves temporal autocorrelation.

Consistent with both groups reporting increased NA after sad mood induction (Zamoscik et al. 2014), the two states were well separable. Gaussian Mixture Models (GMMs) achieved high classification accuracy under shuffled cross‐validation (94% ± 3%) and remained above chance under temporally structured evaluation (67% ± 9%) by assigning each test sample to the condition with higher likelihood (Figure 2b). Comparable performance was obtained with nonlinear support vector machines (SVMs) using a quadratic polynomial kernel (93% ± 3%; 74% ± 10%), whereas linear classifiers such as linear discriminant analysis (LDA) and linear‐kernel SVMs performed poorly under both schemes (30% ± 5%; 44% ± 4%), indicating a nonlinear separation boundary. Logistic regression models with polynomial feature expansions further showed that linear features alone yielded near‐chance performance, whereas inclusion of quadratic terms markedly improved accuracy, with no additional gains from cubic terms (Figure S2), indicating that state separation was well captured by a quadratic decision boundary.

No significant group differences in classification performance were observed between HC and rMDD participants. Given the superior performance of nonlinear classifiers, GMMs were used in downstream analyses to define probabilistic targets for optimal control and to decode neural activity, inferring cognitive states from both empirical and generated trajectories. Neural states remained separable when sampling from the GMMs (Figure S3).

### 2.2 DSR Models Learn the Underlying Dynamics

Next, we inferred subject‐level generative models from the neural data using piecewise linear recurrent neural networks (PLRNNs) trained with a state‐of‐the‐art DSR protocol (Hess et al. 2023). These state‐space type models—also referred to as *world models* in model‐based reinforcement learning (RL; Ha and Schmidhuber 2018; Hafner et al. 2025)—consist of a latent PLRNN model coupled to a linear decoder, enabling the reconstruction of high‐dimensional neural trajectory dynamics (see Section 5.3 for details). Representative reconstructions from the inferred models are shown in Figure 2d.

The inferred dynamics were found to be chaotic, characterized by positive maximum Lyapunov exponents ($\lambda_{\max}=0.1\pm 0.02$), in line with previous findings on neural recordings during resting (Volkmann et al. 2024). This chaotic nature makes it inherently difficult to achieve a point‐by‐point match between predicted and empirical trajectories over time (see also Koppe et al. 2019; Mikhaeil et al. 2022; Schmidt et al. 2021). Despite this, the models successfully captured the data's underlying temporal structure, as evidenced by strong alignment in the recovered frequencies, measured in terms of the Hellinger distance $D_{H}$ between power spectra ($D_{H}=0.22\pm 0.04$, Figure 2e). Moreover, the cross‐correlation between different brain regions—commonly referred to as functional connectivity—exhibited highly similar patterns in both the empirical data and the model‐generated test set trajectories (Figure 2c). These results underscore that, although exact trajectory matching may not be expected, the inferred models nonetheless reproduced the essential statistical and spectral properties of the observed neural activity, indicating successful system identification under the DSR framework (Durstewitz et al. 2023).

### 2.3 Learning Successful Control Policies

Having established our DSR models, we next inferred optimal closed‐loop control policies by augmenting the models with an additive nonlinear state‐dependent control term (see Section 5.3). Here, a closed‐loop control policy refers to an adaptive rule that determines the model input as a function of the current simulated neural state. The DSR model therefore served as a computational surrogate of the empirically observed fMRI dynamics, while the learned control policy specified how this surrogate model would have to be perturbed to guide simulated transitions toward a desired empirical target distribution.

To derive these policies, we fixed the DSR model parameters and optimized only the control input. The objective of this optimization was to steer the inferred systems toward a desired target in a state‐dependent manner. The resulting controlled trajectories are simulated time courses generated by the DSR model while the learned policy is active. In the present analyses, control could be restricted to specific output dimensions, allowing us to ask how virtual modulation of *individual* brain regions steered the reconstructed system from rest to sad mood induction or from sad mood induction back to rest. For example, this allowed us to estimate how modeled input to the amygdala would have to vary with the current neural state for the reconstructed system to reach the sad mood distribution within a specified time horizon.

We considered a *soft target* strategy, which assigns probabilities across states based on the above‐mentioned GMMs and aims to steer the system toward probabilistically representative states (see Section 5.3 for details). To assess the effectiveness of the control policies, we evaluated the negative log‐likelihood (${\mathrm{NLL}}_{\mathrm{Ind}}$ and ${\mathrm{NLL}}_{\text{Rest}}$ for the sad mood induction and resting, respectively) under the GMM. The inferred control policies are governed by two hyperparameters: the control energy regularization parameter $\lambda_{E}$, which penalizes the magnitude of the control input, and the temporal horizon D, which defines the number of time steps allowed for the system to reach the target (cf. Equation (4)).

**FIGURE 3:**
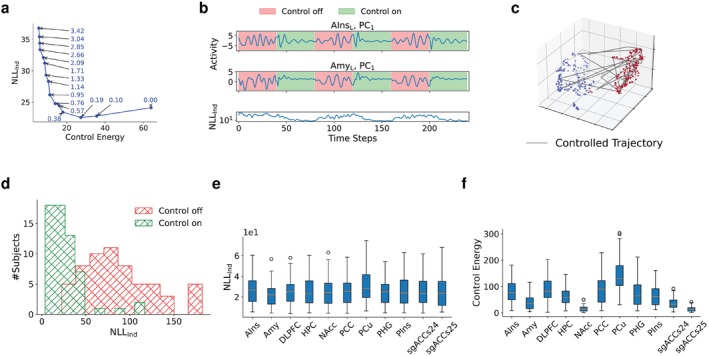
Control successfully steers the dynamics. (a) Pareto front illustrating the trade‐off between ${\mathrm{NLL}}_{\mathrm{Ind}}$ and control energy in a multiobjective optimization, using the amygdala as an example. Corresponding values of $\lambda_{E}$ are indicated in the diagram. Saturation of the control occurred at approximately $\lambda_{E}=10^{-1}$, beyond which less regularization no longer improved ${\mathrm{NLL}}_{\mathrm{Ind}}$. (b) Example trajectories from a controlled region (left Amy; middle) and an uncontrolled region (left AIns; top), both move toward the sad mood induction state, as confirmed by decoding with a Gaussian Mixture Model (bottom). (c) UMAP projection revealed that generated trajectories aligned closely with the empirical sad mood induction state distribution (red), while diverging from resting distributions (blue). (d) Across all 60 subjects, control significantly reduced the NLL of the trajectory over time steps under the GMM. (e) Steering accuracy, quantified as the ${\mathrm{NLL}}_{\mathrm{Ind}}$ target proximity (*y*‐axis), was comparable across all stimulation sites (*x*‐axis), indicating that each region was able to drive the surrogate system equally close to the desired state. (f) In contrast, the control energy (*y*‐axis) required to achieve that steering differed significantly between regions (*x*‐axis).

To determine appropriate parameter values for the control energy regularization term $\lambda_{E}$, we first investigated the effect of $\lambda_{E}$ on the trade‐off between control energy and proximity to the target, for models trained using the soft target approach on the bilateral amygdala (Amy) (Figure 3a) and transitions from resting to sad mood. The resulting curve showed that for sufficiently large (or small) values of $\lambda_{E}$, the optimization favored minimizing either energy or target proximity, respectively. Importantly, the curve exhibited an elbow around $\lambda_{E}=0.1$, beyond which further increases in control energy did not improve target proximity—indicating a balance point in the trade‐off. Based on this observation, we fixed $\lambda_{E}$ at 0.1 for all subsequent analyses. The temporal horizon D was set to 10 to give the network some time to move toward the target using its own dynamics, while still requiring it to reach the target within a reasonable time frame for fMRI. We report results for alternative $\lambda_{E}$ and D in Figure S4, demonstrating that our main conclusions are insensitive to moderate hyperparameter changes.

Figure 3b–e illustrates the efficacy of our control protocol for the case of control towards sad mood induction states, and Figure S5 visualizes control policies across regions for a selected individual. Upon control activation, neural activity converged to the target, reverting to its intrinsic chaotic dynamics once control is deactivated. While state separation became more pronounced when control was applied across multiple brain regions (Figure S6), we observed a significant reduction in target proximity even when single regions were controlled. This improvement was driven by the indirect modulation of non‐controlled regions through network‐level interactions. The control policies also demonstrated the ability to generalize to previously unseen states: although the system could encounter novel configurations during deployment, the policies consistently guided the dynamics back toward the target. In principle, the same procedure could be applied using a hard target approach (Figure S6).

### 2.4 Small Brain Regions Exhibit High Controllability

Next, we assessed controllability. A region is considered more controllable if lower input to that region is sufficient to guide the reconstructed dynamics toward the target state distribution. Thus, controllability refers to the ease of steering the surrogate model (rather than to spontaneous psychological regulation or recovery). With this definition, we focused on two key questions: (1) Can we identify brain regions that are more effective than others in driving state transitions? and (2) How do these effects differ between individuals with rMDD and HC? Using the soft target approach, we derived separate control policies for each bilateral ROI.

While control policies across all regions achieved a comparable proximity to the target (Figure 3e), the amount of energy required to achieve this convergence varied between regions (Figure 3f). Smaller regions, such as the subgenual ACC (sgACC) and nucleus accumbens (NAcc), exhibited enhanced controllability, reaching the target more efficiently. Less controllability in larger brain areas could have arisen because they exhibited more spatial variability (Pearson correlation between size and total variance $r\left(11\right)=0.97$, p<0.001), causing the first principal components (PCs) to have greater variance as confirmed by a strong correlation between brain region size and control energy (Pearson's $r\left(11\right)=0.93$, p<0.001). This increased dynamic range may require greater control energy to steer the distributed activity toward the target. On the other hand, sgACC and NAcc play a central role in negative mood control and are established targets for deep brain stimulation (Figee et al. 2022), such that increased controllability may also be attributed to an emphasized functional role.

### 2.5 Asymmetric Neural Controllability in rMDD: Reduced Energy Costs and Residual Bias Toward Sad Mood States

Next, we examined differences in control policies between rMDD and HC during the transition from resting to sad mood induction states and vice versa.

**FIGURE 4:**
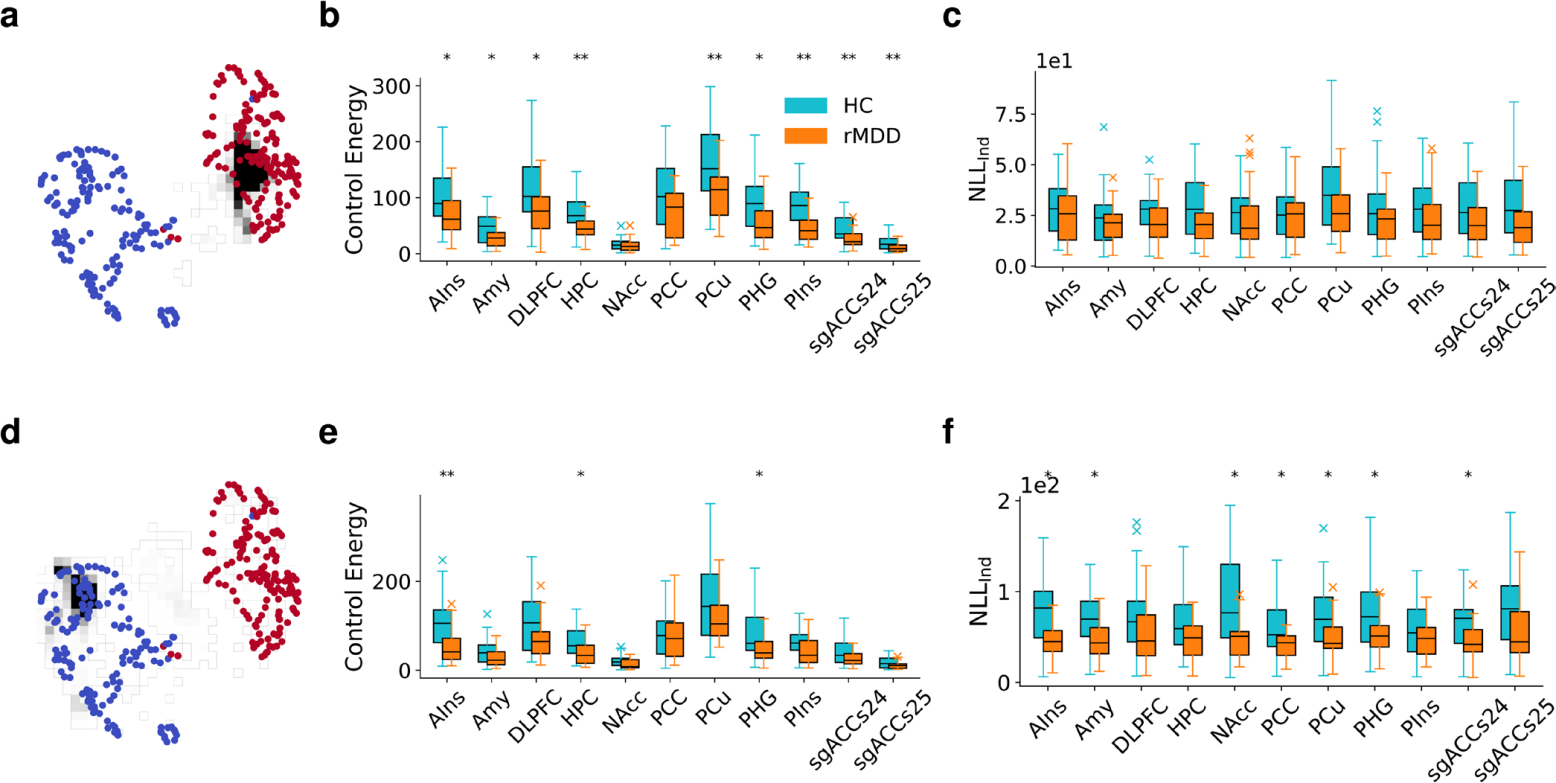
Group control analyses. (a) UMAP visualization of empirical states (blue and red). Different initializations of controlled trajectories yield the heat map (black) of the controlled activity which shows that the activity remains in the sad mood induction region due to the control. (b) Control energy required to drive the brain toward the sad mood state by stimulating individual brain regions. rMDD participants consistently required less energy than HC. (c) Despite the different amounts of energy expended, the groups did not show significant differences in proximity to the sad mood states. (d–f) Same as in (a–c), but for control towards resting. Group differences were assessed using two‐tailed Mann–Whitney U tests across subjects for each region; *p* values were corrected for multiple comparisons across the 11 regional tests using the Benjamini–Hochberg false discovery rate (FDR) procedure. Asterisks denote FDR‐adjusted significance levels: *p_FDR_ < 0.05, **p_FDR_ < 0.01.

During transitions *from resting to sad mood* (Figure 4a), individuals with rMDD required significantly less control energy than HC across nearly all examined regions, with the exception of the NAcc and PCC (Figure 4b). This reduction in energetic cost indicates enhanced neural controllability in rMDD, suggesting that both the transition to and maintenance of the sad mood state were more easily achieved in this population. Importantly, this effect was robust across a broad range of regularization parameters and could not be explained by differences in the separability of baseline proximity between the resting and sad mood states (Figure S4). Despite the energetic differences, no significant group differences were observed in the proximity to the (sad mood) target state as measured by ${\mathrm{NLL}}_{\mathrm{Ind}}$ (Figure 4c), nor to the initial (resting) state (Figure S7).

When transitioning *from sad mood back to resting* (Figure 4d), rMDD participants again showed a tendency toward reduced control energy, though this effect was more spatially restricted, reaching statistical significance only in the anterior insula (AIns), hippocampus (HPC), and PHG (Figure 4e and S8). While both groups approached the target with comparable proximity (Figure S7), rMDD individuals remained closer to the sad mood state (Figure 4f), suggesting a lingering bias toward the sad mood configuration despite reduced energetic demands. This was found to be significant for most regions, apart from the dorsolateral prefrontal cortex (DLPFC), hippocampus, posterior insula (PIns), and parts of sgACC.

### 2.6 Connectivity Predicts Controllability

Finally, we sought to identify mechanistic differences between groups that may underlie the altered controllability patterns observed in rMDD. To this end, we leveraged the piecewise linear architecture of our data‐driven DSR model to estimate *coupling matrices* C, that is, transition dynamics expressed in observation space (see Section 5.3.5 for details).

**FIGURE 5:**
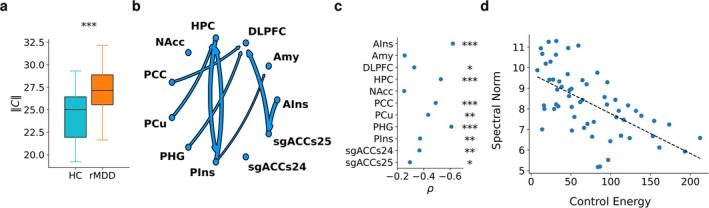
Brain region coupling. (a) Global coupling strength, measured as the spectral norm of the full coupling matrix C, is significantly higher in rMDD. (b) Pairwise coupling strength between brain regions is computed as the spectral norm of 6×6 submatrices. Significant differences between groups in directed coupling ($p_{\mathrm{FDR}}<0.05$), depicted as arrows indicating the direction and strength of influence from one region to another. (c) Correlation between regional coupling strength and control energy. Significant negative correlations indicate that regions with stronger outward influence require less control effort to reach the sad mood states, suggesting a mechanistic link between connectivity and controllability in rMDD. The significance of the correlations was assessed using two‐tailed *t*‐test under the null hypothesis of zero correlation. *p* values were corrected for multiple testing across the 11 regional tests using the Benjamini‐Hochberg false discovery rate (FDR). Asterisks denote FDR‐adjusted significance levels: *p_FDR_ < 0.05, **p_FDR_ < 0.01, and ***p_FDR_ < 0.001. (d) PHG coupling strength (as measured via spectral norm) versus required control energy per subject.

The spectral norm of these coupling matrices, given by the largest singular value, was significantly higher in the rMDD group (Mann–Whitney U = 617, p<0.001, Figure 5a), indicating greater maximal dynamical coupling relative to HC.

To localize these effects, we examined the $\left(6\times 6\right)$ (each region is associated with 2 hemispheres and 3 PCs) submatrices of the full coupling matrices that quantify how activity in one region influences transitions in another. This revealed elevated directed coupling in rMDD, particularly toward—but not away from—the DLPFC (Figure 5b), a region frequently implicated in cognitive control and the top‐down regulation of affect.

Finally, we asked whether regional coupling strength predicted controllability. Specifically, we computed the spectral norm of the 66×6 submatrices representing directed coupling from each region to all others, and correlated these values with the control energy required to reach the sad mood target (Figure 5c,d). Across most regions, stronger outward coupling was associated with lower control energy, suggesting that regions with greater influence over the network facilitate more efficient control. This effect was particularly pronounced in AIns, HPC, PCC, and PHG—all of which showed increased coupling in rMDD, and many of which directly or indirectly modulated DLPFC dynamics (Figure 5b). The correlation was preserved for the reverse direction (Figure S9). These findings may indicate that regions exerting strong outbound influence, especially those interfacing with prefrontal control, play a central role in steering brain state transitions in depression.

### 2.7 Exploratory Associations With Affect and Rumination

In exploratory analyses, we examined whether individual differences in control energy were related to affective ratings and rumination within each group. Within the rMDD group, higher NA reported after the resting session was positively associated with control energy in both transition directions, across multiple regions. The strongest associations were observed in the sgACC (rest to sad mood: r=0.59; sad to rest: r=0.58; $p_{\mathrm{FDR}}<0.05$ across 11 regions per contrast, Table S1). These associations were not observed in HC (all $p_{\mathrm{FDR}}>0.05$).

Rumination was positively associated with control energy specifically for the *sad to rest* transition in rMDD, with the strongest effect in the PHG (r=0.62, $p_{\mathrm{FDR}}<0.05$ across 11 regions, Figure S8, Table S1). This association was not observed for the reverse transition (rest to sad mood) and did not reach significance in HC, although correlations were positive in direction. No significant correlations were found with coupling strength (all $p_{\mathrm{FDR}}>0.05$). Together, these findings indicate that while at the group level the effect of greater controllability leads to less control energy in rMDD patients, individual differences in rumination are associated with increased resistance to disengagement from sad mood states.

These findings should be interpreted with caution. Associations survived correction across brain regions within each affective scale, but none remained significant after joint correction across all tested measures (positive affect, NA, and change scores across conditions). These results are therefore reported as exploratory and hypothesis‐generating.

Finally, to assess whether medication status could account for the observed effects, we performed medication sensitivity analyses. First, within the rMDD group, medicated and unmedicated participants did not differ significantly on the evaluation measures, including coupling strength, control energy, and target proximity (all $p_{\mathrm{FDR}}>0.1$). Second, we repeated the main rMDD–HC comparisons after excluding all medicated rMDD participants. The resulting effect sizes for the remaining unmedicated rMDD participants (n=22) compared with HC were largely consistent with those observed in the full sample (see Table S3), indicating that the overall pattern of rMDD–HC differences was preserved.

## 3 Discussion

This study introduced a data‐driven framework that combines DSR with model‐based control to examine how brain dynamics in remitted rMDD support or resist transitions between emotional states. Our approach leveraged expressive generative models, trained solely on empirical fMRI time series, to reconstruct neural dynamics and enable model‐based causal intervention analyses. By treating these models as computational surrogates of the brain, we extended prior control‐theoretic work—often limited to linear (Deng et al. 2022; Gu et al. 2015; Parkes et al. 2024; Yao et al. 2025) or biophysically constrained (Martínez et al. 2023; Taylor et al. 2015) models—into a generalizable and expressive machine learning framework capable of simulating and controlling neural surrogate trajectories at the level of individual subjects.

Unlike traditional correlational analyses, our framework provides a causal account of how neural perturbations drive transitions between functional states. By learning optimal control policies on reconstructed latent dynamics, we can identify how activating or deactivating specific brain regions shapes trajectories of neural activity. We illustrated this approach by examining mechanisms through which individuals with rMDD more readily enter, sustain, or exit states of sad mood, highlighting potential intervention targets for modulating pathological state transitions.

### 3.1 Validity of the Generative Models

Central to our approach was the training of DSR models that accurately reconstruct empirical neural dynamics. Beyond capturing the complex temporal evolution of the data, the models replicated key statistical and connectivity‐based properties. In particular, functional connectivity patterns in the model‐generated trajectories closely mirrored those observed in the empirical recordings. Notably, the models revealed elevated directed coupling in rMDD participants, consistent with previous findings of altered network interactions in depression (Lydon‐Staley et al. 2019; Zhu et al. 2012), and elevated connectivity observed specifically in this dataset (Zamoscik et al. 2014), although our results were derived entirely from a data‐driven approach. This cross‐methodological convergence supports the robustness of both our modeling framework and its capacity to extract clinically relevant neural signatures.

### 3.2 Reduced Control Energy, Persistent Sad Mood Dynamics in rMDD, and Within‐Group Heterogeneity

Our primary finding indicated that individuals with rMDD require significantly less control energy to transition into and maintain sad mood states compared to HC. This reduced energetic cost suggests that, at the group level, the reconstructed dynamical landscape in rMDD is shaped in a way that favors transitions toward sad mood states, aligning with prior reports of heightened negative and diminished positive affect (PA) in response to sad mood induction in this group (Zamoscik et al. 2014). We also found that this energetic advantage was widespread across regions, with only a few exceptions. Such global efficiency implies that the facilitation is not localized to a single structure but emerges from network‐level interactions.

More unexpectedly, rMDD participants also required less energy than controls to transition from the sad mood state back to rest (although this effect was far less pronounced across regions). This might appear inconsistent with the idea of heightened vulnerability; however, a closer look revealed an important asymmetry. Although both groups could be guided toward the resting state distribution under active control, rMDD trajectories *remained closer to the sad mood state* even while control was still applied. This implies that, in rMDD, even when the system is successfully pushed toward rest, its underlying dynamics continue to partially align with the sad mood configuration. The models therefore indicated a form of incomplete disengagement: the system can move toward rest efficiently, yet its intrinsic dynamics continue to partially align with the sad mood configuration. This pattern suggests that sad mood configurations may remain partially stabilized once entered, potentially contributing to the well‐documented difficulty rMDD individuals have in sustaining positive or neutral affect even after recovery from depressive episodes (Moulds and McEvoy 2025; Paykel 2008; Timm et al. 2017).

Importantly, exploratory analyses further revealed meaningful heterogeneity within the rMDD group. Higher NA reported after the resting state was associated with increased control energy in both transition directions, particularly in the sgACC. This finding is also plausible in light of previous work linking subgenual cortical coupling to the tracking of self‐blame, a specific self‐evaluative process associated with NA in rMDD (Green et al. 2012; Lythe et al. 2022). It may suggest that sgACC is not just correlated with NA but may help organize how maladaptive affective states are maintained and re‐entered in depressive states. Moreover, higher levels of daily‐life rumination were associated with greater control energy specifically for disengagement from induced sadness (sad mood to rest), most prominently in the PHG. Although these associations did not survive correction across all tested affective scales and must therefore be interpreted cautiously, their direction is theoretically coherent: individuals with elevated cognitive‐affective vulnerability require greater external input to shift neural dynamics away from sad mood.

Taken together, these findings suggest a nuanced picture. At the group level, rMDD was characterized by globally altered dynamics that make affective state transitions energetically cheaper. At the individual level, however, higher rumination indices increased resistance to disengagement from negative states.

### 3.3 Connectivity as the Mechanistic Link

The reduced control energy in rMDD compared to HC was strongly predicted by increased coupling strength across the network. Regions with stronger outbound influence over others required less external input to drive the whole system toward the target. This finding provides a mechanistic bridge between controllability and network structure: highly connected regions act as natural leverage points that can efficiently propagate state changes. It is also consistent with the observation that networks with higher mean degree not only need fewer driver nodes but also incur lower control energy, because the extra connections provide multiple, “low‐resistance” pathways for steering the system (Liu et al. 2011). Interestingly, we observed elevated coupling especially directed toward (and not away from) the DLPFC—a region implicated in cognitive control and emotion regulation (e.g., Nejati et al. 2021; Vanderhasselt et al. 2013).

An alternative interpretation of our findings is that heightened coupling reflects a compensatory reorganization in rMDD. In our exploratory analyses, higher rumination was associated with greater control energy for the transition from sad mood induction back to rest, suggesting that individuals with stronger residual cognitive vulnerability may be more resistant to disengaging from sad mood states. Against this background, increased coupling could represent a countervailing transition‐facilitating mechanism, whereby stronger coupling reduces the energy required to move between neural states. Such a mechanism could be adaptive if it supports flexible movement away from maladaptive states or helps stabilize functioning after remission. At the same time, in the present sad mood induction paradigm, this same transition‐facilitating property may have mixed consequences. Stronger coupling may make sad mood states easier to reach and may also contribute to the residual proximity to the sad mood distribution observed during return‐to‐rest. The present cross‐sectional design cannot determine whether heightened coupling reflects vulnerability, compensation, resilience‐related adaptation, or a combination of these processes.

### 3.4 Outlook

Our findings have at least two implications. First, they offer a mechanistic explanation for the increased vulnerability to sad mood in depression: the brain's intrinsic dynamics make transitions into sadness easy and recovery only partially complete, even under active control. Second, they suggest putative causal targets for intervention. Our analyses revealed strong negative correlations between control energy and coupling strength in multiple brain regions examined, but particularly the HPC, the PHG, and the AIns—regions already implicated in affective control and neuromodulation therapies (Arulchelvan and Vanneste 2023; Liu et al. 2021; Roet et al. 2020). Moreover, small regions like sgACC and NAcc were confirmed as particularly efficient hubs facilitating transitions between resting and sad mood states, emphasizing their role as efficient control points for modulating maladaptive dynamics in depressive disorders.

Beyond theoretical insight, our framework offers a principled way to quantify regional controllability within reconstructed neural dynamics. Because control is learned separately for each individual brain region, the method enables systematic model‐based perturbation analyses of potential stimulation targets, identifying regions that can steer the system with minimal energy or maximal precision (see also Fechtelpeter et al. 2026; Luo et al. 2025; Muldoon et al. 2016).

At the same time, it is important to clearly distinguish between computational control within the reconstructed model and biophysical implementation in the brain. Similarly, controllability should not be equated with spontaneous mood recovery or psychological regulation. Biological systems may return from sad mood toward resting states through intrinsic dynamics and regulatory processes, without external perturbation. The inferred control inputs, instead, represent abstract perturbations in model space derived from BOLD time series and cannot be interpreted in physical units. Translation to neuromodulatory or pharmacological interventions would require empirical characterization of how such interventions reshape the underlying neural dynamics and how these effects map onto the reconstructed state space.

Nevertheless, the framework conceptually aligns with emerging efforts toward adaptive and personalized neuromodulation (Santaniello et al. 2011; Steffen et al. 2024). By providing a mechanistic characterization of how specific regions influence affective state transitions, it may inform hypothesis‐driven selection of candidate targets or guide future experiments that explicitly couple stimulation data with dynamical system identification. In this sense, the present work should be understood primarily as offering mechanistic and theoretical insight, while outlining a computational scaffold that could, in principle, be extended toward translational applications.

### 3.5 Limitations

Despite these coherent findings, the control approach remains to be validated. First, associations between control energy and behavioral measures were exploratory and did not survive correction across all tested affective scales. These findings therefore require independent replication before firm conclusions about clinical validity can be drawn. Likewise, while medication sensitivity analyses did not indicate that medication status accounted for the main model‐derived effects, the medicated rMDD subgroup was small. The present analyses therefore cannot exclude more subtle medication‐related influences on reconstructed neural dynamics, control energy, or coupling strength.

Second, the present findings are derived from a single, well‐characterized dataset using a specific sad mood induction paradigm in individuals with rMDD. The stability of reconstructed dynamics and controllability patterns across repeated sessions was not assessed, leaving open the question of whether control energy reflects a trait‐like property or fluctuates with transient affective states. Moreover, different affective manipulations—such as stress induction, autobiographical recall without music, or positive mood induction—may induce distinct geometries of neural state space and therefore yield different controllability profiles. It also remains unclear whether currently depressed individuals would exhibit quantitatively amplified or qualitatively altered patterns.

Third, our inferences are based entirely on model‐based simulations of reconstructed dynamics derived from BOLD signals. The inferred control inputs represent abstract perturbations in model space and do not incorporate the physics of electromagnetic stimulation, pharmacokinetics, or neurovascular coupling. Accordingly, the term control energy refers to the mathematical cost term used during model optimization and should not be interpreted as physiological energy consumption or metabolic demand. Consequently, the reported control energies cannot be directly translated into specific stimulation parameters or pharmacological dosages. Real‐world neuromodulatory interventions would require empirical characterization of how stimulation reshapes neural dynamics and how these effects map onto the reconstructed state space. Future work should therefore (i) examine the reliability of control metrics across sessions, (ii) test their generalizability across different affective paradigms and clinical states, and (iii) integrate empirical stimulation data to bridge the gap between computational control and biophysical implementation.

More generally, the quantities reported here should be understood as model‐based constructs rather than direct empirical observables. They arise from applying a control‐theoretic probe to dynamics reconstructed from empirical fMRI time series. The central object of inference is therefore the learned dynamical surrogate, while the controller provides one formal way to interrogate how these dynamics can be steered between empirically defined state distributions. The specific controller parameterization is not unique: alternative control formulations could in principle be applied to the same reconstructed dynamics, and the control objective could also be transferred to other DSR approaches or generative models of neural dynamics. Ideally, sufficiently expressive and well‐validated DSR models should recover similar qualitative dynamical structure from the same empirical data, in which case controllability profiles would be expected to show corresponding robustness. Future work should test this explicitly by benchmarking controllability profiles across model classes and controller parameterizations.

## 4 Conclusion

In summary, our study bridges the gap between dynamical systems theory, clinical neuroscience, and RL‐based control by providing a data‐driven framework to investigate neural state transitions and how they could be manipulated. The altered controllability and connectivity patterns in rMDD highlight the potential of this approach to uncover novel neural dynamics and therapeutic targets for mood disorders.

## 5 Methods

### 5.1 Sample

We reanalyzed a data set first reported in Zamoscik et al. (2014) (see also Huffziger et al. 2013; Lydon‐Staley et al. 2019; Timm et al. 2017; Zamoscik et al. 2018; Zhang et al. 2023 for elaborate details). In brief, the study included 30 individuals with rMDD—defined as having experienced at least two episodes of MDD—and 30 HC matched for age, sex, and education, with no history or current diagnosis of MDD. The rMDD group had to be in partial or full remission for a minimum of 2 months. Exclusion criteria for all participants included clinical diagnoses of bipolar disorder, psychotic disorders, substance dependence, current substance abuse, generalized anxiety disorder, obsessive‐compulsive disorder, post‐traumatic stress disorder, eating disorders (all as per DSM‐IV), and contraindications for MRI. A trained clinical psychologist conducted the diagnostic assessments using the Structured Clinical Interview for DSM‐IV Axis I. Medication status was recorded for the rMDD group at the time of scanning. About 8 of the 30 rMDD participants were receiving psychotropic medication. When participants on psychotropic medication were excluded from the analysis, the main findings on brain connectivity and symptom prediction still remained, although some associations were no longer statistically significant because the sample became smaller (Zamoscik et al. 2014). The study received approval from the local ethics committee of Heidelberg University and conformed to the Declaration of Helsinki. All participants provided written informed consent.

### 5.2 Experimental Data

Based on the study by Zamoscik et al. (2014), we selected two 4.5‐min fMRI sessions for analysis: the first resting state and sad mood induction session from their more complex experiment. Sad mood induction was achieved using three personally significant negative life events, identified immediately prior to the fMRI session (e.g., a breakup, the death of a loved one, or a traumatic experience). These events were presented sequentially as keywords (each displayed for 1.5 min) accompanied by instrumental background music (excerpts from Albinoni's Adagio in G minor). Mood assessments were conducted using the Positive and Negative Affect Scale (PANAS; Watson et al. 1988) before and after the sad mood induction. Both groups reported sadder mood after mood induction where rMDD reported a significantly more increased NA and a similarly decreased PA compared with HC (Zamoscik et al. 2014). During the resting state session, participants also viewed emotionally neutral words (namely, “rest 1,” “rest 2,” “rest 3”).

#### 5.2.1 Preprocessing

The study used a 3T Trio Tim Scanner with a 12‐channel head coil (Siemens Healthineers, Erlangen, Germany), acquiring functional images at a rate of 1.5 s (TR). For additional details on data acquisition, data processing, and artifact correction, see Supporting Information: “Data Preprocessing and Artifact Correction.”

To obtain time‐series data for modeling, anatomical masks were used to extract 11 brain regions from both hemispheres (Figure 2a). These regions were chosen to provide system‐level coverage of five large‐scale networks consistently implicated in MDD—salience/limbic, default mode, cognitive control, reward, and memory networks—based on converging evidence from meta‐analytic studies showing structural and functional alterations in these circuits (Gou et al. 2023; Kaiser et al. 2015; Ng et al. 2019; Schmaal et al. 2016).

Time series were standardized and subsequently bandpass filtered using cutoff frequencies of 0.0083 and 0.15 Hz. For each subject, data were projected onto the top three PCs, which explained an average of 76% of the variance, with a minimum of 50% across all subjects. This yielded a 66‐dimensional time series (2 hemispheres × 11 regions × 3 PCs), comprising 320 time points per subject, with 160 time points for each of the resting state and mood induction conditions. Finally, the time series—a time point of which is denoted as $x_{t}$ in the following—were standardized for PLRNN, SVM, and GMM training. This scaling was reversed during control optimization, restoring each dimension to its original variance.

### 5.3 DSR Model and Control

#### 5.3.1 DSR Model Architecture

To model the dynamics within the selected ROIs, we employed a state‐space framework comprising a latent dynamical model coupled with an observation model. The latent dynamics were governed by the shallow PLRNN (shPLRNN; Hess et al. 2023), defined as follows:

(1) $$z_{t+1}={\mathrm{Az}}_{t}+W_{1}\left(\sigma \left(W_{2}z_{t}+h_{2}\right)-\sigma \left(W_{2}z_{t}\right)\right)+h_{1}$$

with latent states $z_{t}\in {\mathbb{R}}^{M}$, diagonal matrix $A\in {\mathbb{R}}^{M\times M}$, connectivity matrices $W_{1}\in {\mathbb{R}}^{M\times L}$ and $W_{2}\in {\mathbb{R}}^{L\times M}$, and biases $h_{2}\in {\mathbb{R}}^{L}$ and $h_{1}\in {\mathbb{R}}^{M}$, where L is the dimension of the hidden layer, and $\sigma \left(\cdot \right)$ is an elementwise piecewise linear activation function (the Rectified Linear Unit; ReLU).

The observation model was linear and defined as $x_{t}={\mathrm{Bz}}_{t}$, where $B\in {\mathbb{R}}^{O\times M}$ and O=66 is the observation dimension.

Each model was trained jointly on both the resting state and sad mood induction data from a single subject, without access to condition labels during training.

#### 5.3.2 Model Training

Models were trained using backpropagation through time (BPTT) combined with generalized teacher forcing (GTF; Hess et al. 2023), a technique in which model‐generated latent states are partially realigned with encoded states after gradient computation using linear interpolation with the interpolation parameter decaying from $\alpha_{0}$ to $\alpha_{1}$ during training. This alignment helps mitigate exploding gradients during training. Encoded latent states $z^{\mathrm{enc}}$ were obtained by projecting observations into the latent space via the Moore‐Penrose pseudoinverse $B^{+}$.

Training was performed by minimizing a regularized mean squared error loss, which penalizes discrepancies between model‐generated and empirical observations, as well as between model‐generated and encoded latent states,

(2) $$\begin{array}{c} \begin{array}{l} L_{\mathrm{DSR}}=\frac{1}{\text{TO}}\sum_{t=1}^{T}∥x_{t}^{\mathrm{emp}}-x_{t}^{\mathrm{gen}}{∥}^{2}+\frac{1}{\mathrm{TM}}\sum_{t=1}^{T}∥z_{t}^{\mathrm{enc}}-z_{t}^{\mathrm{gen}}{∥}^{2}\\ +\lambda_{1}{\left(1-\kappa \left(B\right)\right)}^{2}+\lambda_{2}∥I-A{∥}_{F}^{2}+\lambda_{3}∥W_{1}{∥}_{F}^{2}+\lambda_{4}∥W_{2}{∥}_{F}^{2}, \end{array} \end{array}$$

where $x_{t}^{\mathrm{gen}}$ and $z_{t}^{\mathrm{gen}}$ refer to model generated observations and latent states generated according to Equation (1) and the observation model, and $\kappa \left(B\right)$ denotes the condition number of the observation matrix B that is regularized towards 1 to promote numerical stability of the pseudoinverse via $\lambda_{1}$. The $\lambda_{2}$‐term regularizes the diagonal entries of A toward 1 and $\lambda_{3}$ and $\lambda_{4}$ regulate $L_{2}$ penalties on $W_{1}$ and $W_{2}$, $∥\cdot {∥}_{F}$ denotes the Frobenius norm.

We used the RAdam optimizer (Liu et al. 2020), with an exponentially decaying learning rate from $\eta_{0}$ to $\eta_{1}$, and momentum parameters $\beta_{1}=0.9$ and $\beta_{2}=0.999$.

The models were trained on the first 120 time steps of each session (75%; training set) and evaluated on the remaining 40 time steps (test set). During each training epoch, a full batch of 162 sequences of length T=40 was sampled—comprising 81 sequences from the 120 training time steps of both the resting state and sad mood induction sessions. The hyperparameters including the matrix dimensions used in training were selected via grid search on the test set for a subset of five subjects, with the final values summarized in Table S1. For each participant, we trained five models with Xavier initialization (Glorot and Bengio 2010) for up to 200,000 epochs and selected the model with the lowest test loss for subsequent experiments.

#### 5.3.3 DSR Model Evaluation

To quantify how well the DSR model reproduces the temporal structure of the data, we compared the power spectrum of each latent dimension between the measured trajectory and the model's reconstruction along the entire time series. Concretely, for each dimension $i\in \left\{1,\ldots ,O\right\}$ we computed the Fast Fourier transform of the time series (Cooley and Tukey 1965), smoothed the resulting power spectrum with a Gaussian kernel, and renormalized it to obtain two spectral densities, $S_{i}\left(\omega \right)$ (measured) and $P_{i}\left(\omega \right)$ (reconstructed). We then summarize their discrepancy by the mean Hellinger distance, $D_{H}\left(S,P\right)=\frac{1}{O}\sum_{i=1}^{O}\sqrt{1\;-\;\int_{-\infty }^{\infty }\sqrt{\;S_{i}\left(\omega \right)\;P_{i}\left(\omega \right)\;}\;\mathrm{d\omega}}.$ This metric ranges from 0 (identical spectra) to 1 (maximally dissimilar), and thus provides a dimension‐averaged measure of how faithfully the shPLRNN captured the frequency content of the original signals.

To further assess model performance on unseen data, we also compared the functional connectivity patterns in the empirical and reconstructed test‐set trajectories. Specifically, we computed the Pearson correlation matrix across all observed regions for both the empirical data and the held‐out shPLRNN reconstructions. This connectivity comparison ensured that the shPLRNNs not only reproduce spectral properties but also preserve the inter‐regional coupling structure present in the test data.

#### 5.3.4 Model‐Based Control

Our framework is an RL strategy that leverages a model of the system to predict future dynamics and optimize control actions over a finite time horizon. Instead of estimating a value function that describes the expected return from a state, our framework implicitly evaluates states by running forward simulations. We integrated control with DSR to determine the stimuli required to steer the brain into pre‐determined desired states (Figure 1). Using shPLRNNs (Equation 1) trained on empirical fMRI data, we defined an additive, state‐dependent control term $u\left(z_{t}\right)$ that modifies the latent dynamics as follows

(3) $$z_{t+1}=\text{shPLRNN}\left(z_{t}\right)+u\left(z_{t}\right),\;u\left(z_{t}\right)≔K_{2}\sigma \left(K_{1}z_{t}+b\right),$$

where $\sigma \left(\cdot \right)=\text{ReLU}$, $K_{1}\in {\mathbb{R}}^{Q\times M}$, $K_{2}\in {\mathbb{R}}^{M\times Q}$ and $b\in {\mathbb{R}}^{Q}$. The shPLRNN parameters are fixed post‐training; only the control parameters $\left(K_{1},K_{2},b\right)$ are optimized (Figure S1).

##### 5.3.4.1 Soft Target Objective

To allow flexible control, we defined a soft target using a GMM trained on empirical resting state or sad mood induction data (see Section 5.3.6). Instead of reaching a fixed state, the controlled trajectory is guided into high‐likelihood regions under the GMM.

The loss function thus consists of a negative log‐likelihood term and a regularization term penalizing control energy:

(4) $$\begin{array}{c} L_{\text{cont}}=-c\sum_{t=D+2}^{T}\log p_{\mathrm{GMM},s}\left(x_{t}^{\mathrm{gen}}\right)+\lambda_{E}E_{\text{cont}}, \end{array}$$

where $c=\frac{10^{-4}}{T-D-1}$ ensures loss terms are comparable in magnitude, and control energy is defined as

(5) $$E_{\text{cont}}=\sum_{t=1}^{T}∥s⊙\mathrm{Bu}\left(z_{t}\right){∥}^{2},$$

where s are the pre‐standardization standard deviations, restoring the original scale of each PC. Control energy is therefore a mathematical measure of the squared magnitude of the model input required to steer the reconstructed dynamics. It quantifies modeled intervention effort within the surrogate system and should not be interpreted as physiological or metabolic expenditure in the biological brain.

##### 5.3.4.2 Control Optimization

Control parameters $K_{1},K_{2},b$ were optimized using the RAdam optimizer over up to 30,000 epochs with a constant learning rate η. Each trajectory is initialized from an empirical state and runs for T=D+11 steps (1 for initialization, D before loss evaluation, and 10 to maintain the target). We used 240 empirical states for training and 80 for testing. Hyperparameters were set to $\lambda_{E}=0.1$, Q=17, and η=0.01. Among five independently trained models, the one with lowest test loss was selected for analysis. The optimization was robust to initialization (Figure S12).

##### 5.3.4.3 Restricting Control to Specific Brain Regions

To assess controllability and obtain interpretable region‐specific influence, we constrained the control to act only on selected brain regions. While latent space constraints are trivial (e.g., via L1‐regularization to promote sparsity), enforcing constraints in observation space is more complex due to the linear observation model, where latent dimensions are mixed and do not map directly onto individual brain regions.

We overcome this by projecting $u\left(z_{t}\right)$ onto the null space of the decoder rows corresponding to regions where control should be inactive. Let $B_{\mathrm{red}}\in {\mathbb{R}}^{M_{\mathrm{red}}\times M}$ denote the submatrix of B corresponding to these regions. We compute an orthonormal basis $N\in {\mathbb{R}}^{M\times \dim\;\left(\ker\;B_{\mathrm{red}}\right)}$ of ker($B_{\mathrm{red}}$) via singular value decomposition and construct the projection matrix $P={\mathrm{NN}}^{⊤}$ (see also Fechtelpeter et al. 2026). The constrained dynamics then become

(6) $$z_{t+1}=\text{shPLRNN}\left(z_{t}\right)+\mathrm{Pu}\left(z_{t}\right).$$

This ensures that $\mathrm{Bu}\left(z_{t}\right)$ has no component in the restricted observation dimensions. The projection matrix P was computed once before training of the controller and remained fixed throughout optimization.

#### 5.3.5 Coupling Matrix and Lyapunov Spectra

The shPLRNNs (Equation 1) permit efficient computation of coupling matrices and Lyapunov spectra along individual trajectories by exploiting the model's Jacobians $J_{z_{t}}$. At latent state $z_{t}$, the Jacobian of the update map $z_{t}\mapsto z_{t+1}$ is

(7) $$J_{z_{t}}={\left.\frac{{\mathrm{dz}}_{t+1}}{{\mathrm{dz}}_{t}}\right|}_{z_{t}}=A+W_{1}\left(D_{W_{2}z_{t}+h_{2}}-D_{W_{2}z_{t}}\right)W_{2}\equiv A+W_{z_{t}}$$

where

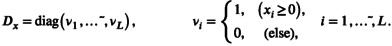

Here, $W_{z_{t}}$ captures how different latent dimensions interact at time t, and is often called the cross‐connection matrix.

Since our observations $x_{t}$ are related to $z_{t}$ by the linear map $x_{t}=B\;z_{t}$, the corresponding Jacobian in observation space is

$$\frac{\partial x_{t+1}}{\partial x_{t}}\;=\;B\;\left(A+W_{z_{t}}\right)\;B^{+}\;\equiv \;C.$$

In other words, C is the coupling matrix of the observable dynamics. We thus interpret ∥C∥ (its spectral norm) as quantifying how strongly activity in one region influences activity in another. We also evaluated ∥C∥ at every $z_{t}$ visited by the shPLRNN when driven by empirical resting state and sad mood‐induction data, yielding a time‐resolved measure of inter‐regional coupling.

To compute Lyapunov exponents, we applied the algorithm of Vogt et al. (2022), which orthogonally projects $A+W_{z_{t}}$ onto tangent subspaces along each trajectory.

#### 5.3.6 Statistical Analysis

##### 5.3.6.1 State Separation

To estimate the empirical distribution of brain states during resting state and sad mood induction, we trained separate GMMs of the respective datasets per subject. GMMs provide a probabilistic description of the state‐space, capturing regions of high empirical density while allowing generalization to nearby states. Model selection based on the Bayesian Information Criterion (BIC) and Akaike Information Criterion (AIC) indicated that six mixture components best described the data (Figure S3).

To validate separability and characterize the form of the decision boundary between resting and sad mood states, we additionally compared linear and nonlinear classifiers (LDA, linear‐ and nonlinear‐kernel SVMs, and $L_{2}$‐regularized logistic regression with polynomial feature expansions). Logistic regression models with linear, quadratic, and cubic feature expansions were used to determine the degree of nonlinearity required to capture separability. Classification was performed separately for each subject on the 66‐dimensional PCA‐reduced time series.

Performance was evaluated using two variants of five‐fold cross‐validation: (i) shuffled folds, in which time points were randomly assigned to folds to assess separability in state space, and (ii) temporally structured folds, in which contiguous time segments were assigned to folds to preserve autocorrelation and assess generalization across time. For downstream control analyses, the final GMM defining target distributions was trained on the full dataset per subject.

To quantify group‐level differences in brain state distributions, we computed three distances between resting and sad mood GMMs: (i) maximum mean discrepancy (MMD), (ii) the $L^{2}$ norm in the space of square‐integrable functions (both computed in closed form), and (iii) the sliced Wasserstein distance (estimated via sampling).

##### 5.3.6.2 Control Evaluation and Group Statistics

For model evaluation under control, trajectories were initialized from each of the 160 empirical brain states (resting or sad mood induction). After an initial warm‐up period of D steps, we computed both the NLL under the target GMM and the cumulative control energy.

To assess regional specificity, the control experiment was repeated 11 times—each time with control restricted to one bilateral region. Across all 30 subjects per group and 160 initializations per condition, the resulting trajectories span T=80 time steps, yielding NLL and energy tensors of shape $\left(30,160,T\right)$. For statistical analysis, the first D steps were excluded to limit analyses to time points where target distance was penalized during training; the median was then computed across time and trials to yield a single value per subject.

Outliers were excluded based on the 1.5× interquartile range criterion. Unless otherwise specified, statistical tests were two‐tailed with a significance threshold of α=0.05. Correlations were assessed using Pearson correlation. Group differences were assessed using Mann–Whitney U tests. False discovery rate (FDR) correction using the Benjamini–Hochberg procedure was applied separately across brain regions for each family of related comparisons (e.g., regional controllability analyses). Exact *p* values and correction status, if applicable, are reported in the Results, and labeled with p or $p_{\mathrm{FDR}}$, respectively.

##### 5.3.6.3 Behavioral and Clinical Measures and Exploratory Brain‐Behavior Analyses

To relate model‐derived controllability metrics to behavioral and clinical variability, we considered (i) affect ratings acquired during scanning and (ii) ambulatory measures of rumination collected outside the scanner as part of the original study protocol (Zamoscik et al. 2014).

Affective state during scanning was assessed with the PANAS administered before and after each fMRI phase. For the present analyses, we used NA and PA ratings obtained after the resting phase and after the sad mood induction phase, and associated change scores, amounting to six tested scales.

Rumination in daily life was assessed using ambulatory assessment over two consecutive weekdays with 10 pseudo‐randomized prompts per day. At each prompt, momentary ruminative self‐focus was assessed using two items, which were averaged and then aggregated across prompts to obtain a subject‐level rumination score (Zamoscik et al. 2014).

Exploratory brain—behavior analyses tested associations between individual differences in control energy (median across time and initializations per subject, separately for each transition direction and stimulation site) and these behavioral measures. In addition, we examined exploratory associations between behavioral measures and regional coupling strength derived from the reconstructed dynamics (Section 5.3.5). Correlations were quantified using Pearson's r and assessed using the standard two‐tailed test for zero correlation. Multiple comparisons were controlled using the FDR procedure across regions within each family of tests (e.g., control energy—rumination correlations across the 11 stimulation sites, separately per transition direction), but not across the number of tested scales. All exploratory analyses are clearly labeled as such and are interpreted cautiously.

## Funding

This work was funded by the Federal Ministry of Research, Technology and Space (BMFTR) under the neuroAI initiative (01GQ2509B), by the German Research Foundation within the Research Unit FOR 5159 (subproject 11), within the framework of the Excellence Strategy of the German Federal and State Governments, by the Wellcome Trust (FUTURE‐D, Z334349/Z/25/Z), and by the Hector II foundation.

## Supporting information


**Figure S1:** Closed‐loop Control Framework. shPLRNNs inferred on the data model the uncontrolled brain dynamics (center). Control is applied via an additive term $u_{t}=u\left(z_{t}\right)$ (bottom left), optimized to drive the system toward a target state while minimizing control effort (top left). To restrict control to specific brain regions, ${\tilde{u}}_{t}$ is projected onto the null space of corresponding rows of the decoder *B*, using the projection matrix ${\mathrm{NN}}^{⊤}$ (bottom right).
**Figure S2:** Indication of Nonlinear Separation Between Conditions. (a) To test the contribution of nonlinear information, we trained L2‐regularized logistic regression models on degree‐3 polynomial feature expansions while progressively increasing the number of included terms. Feature sets were built incrementally from linear terms first, then by adding quadratic and cubic interaction terms. Mean test accuracy and standard deviation across subjects are shown as a function of feature count. Performance increases with the addition of quadratic features, indicating the presence of nonlinear structure, but decreases again when cubic terms are included, consistent with overfitting. (b) The zoomed‐in plot illustrates that linear features are not sufficient to obtain accuracies that differ from chance level.
**Figure S3:** (a) UMAP dimensionality reduction applied to states sampled from the Gaussian Mixture Models (GMMs) confirms that the empirical separation is well preserved (cf. Figure 2b). (b) Model selection using AIC and BIC across subjects suggests optimal cluster number of 6. (c) Group comparisons of the distance between GMMs fitted to the resting and sad mood induction states. No significant differences were observed across metrics: L2 Distance (p = 0.81), Maximum Mean Discrepancy (MMD, p = 0.92), and Sliced Wasserstein Distance (SW, p = 0.74).
**Figure S4:** Robustness to Variations in Control Energy Regularization λ_E_ and Time Horizon D for Control toward Sad Mood. (a) Without an explicit penalty on control energy (λ_E_ = 0), overall energy levels rise, and the rMDD group generally requires less control (statistically significant within HPC and precuneus; PCu). (b) With a shorter horizon (D = 5), the model has fewer time steps before penalties on control energy and target distance apply; rMDD likewise requires less control energy. (a, b) *pFDR < 0.05.
**Figure S5:** (a–k) Two‐dimensional UMAP visualizations of empirical states (sad mood induction in red, resting in blue) and trajectories (black) controlled towards sad mood for different targeted brain regions in a fixed subject. The control successfully maintains activity within the sad mood induction state‐space region. Trajectories from multiple initializations are aggregated by spatial binning, and relative frequencies per bin are displayed as a heatmap. Time steps smaller than D = 10 were discarded.
**Figure S6:** Hard Target Control: A specific sad mood state (red dashed line) was designated as the target, and control was optimized using mean squared error (MSE) loss. Control was applied to all cortical regions. (a) When control is activated, trajectories reliably converge toward the target state (red dashed line); when deactivated, dynamics revert to chaotic behavior. This effect is visible in both controlled (right AIns) and uncontrolled (left NAcc) dimensions. The SVM decoder confirms convergence into the resting state region. (b) UMAP visualization of a trajectory initialized at sad mood induction (red) converging to the target resting state (blue). (c) Control significantly reduces the Euclidean distance to the target state (median across time), consistently across all subjects.
**Figure S7:** No Group Differences in Terms of Proximity to Resting States: Both for the transition from resting state to sad mood (a) and sad mood to resting state (b), rMDD and HC maintain a similar proximity to the resting state.
**Figure S8:** Correlation Between Rumination Ratings and Control Energy. In rMDD patients, higher rumination ratings are associated with increased control energy required to drive neural activity from sad mood states toward resting states. Shown is control applied to the parahippocampal gyrus (PHG) (r = 0.62, pFDR < 0.01).
**Figure S9:** Correlation for Transitions Toward Resting State. (a) For the control in the resting direction, the negative correlation between region coupling and control energy again shows that increased coupling strength is associated with regions with reduced energy expenditure. (b) Specific values of the spectral norm and the energy for each subject on the example of the PHG (c.f. Figure 5c,d).
**Figure S10:** Temporal Evolution of the Evaluation Measures. Control energy (a), NLL_Rest_ (b), and NLL_Ind_ (c) for the case of amygdala‐targeted control. Curves show the median across subjects and initializations; shaded areas denote the interquartile range. While rMDD subjects consistently require less control energy, no significant differences are observed in the likelihoods that quantify the proximity to initial and target states.
**Figure S11:** Connectivity Differences When Training was Performed on Data Corrected for Potential Physiological Artifacts. When correcting the data for physiological effects (i.e., cardiac and respiratory noise) before training the shPLRNNs, the significant difference in connectivity between the groups remains evident. Both the global coupling (a) and the pairwise connectivity between brain regions (b, difference in median, rMDD‐HC) are significantly stronger in rMDD. Here, *pFDR < 0.05, **pFDR < 0.01, ***pFDR < 0.001.
**Figure S12:** Robustness of Control Parameters to Initialization. For each experiment and subject, five models were independently optimized. Controlled trajectories show predominantly consistent control inputs u_t_ (a) and corresponding activity x_t_ (b) across runs, each represented by a separate color‐coded curve, displayed for a selected number of subjects. While the chaotic dynamics amplify small deviations over time, variance across runs of control energy (c) and NLL_Ind_ (d) remains substantially smaller than inter‐subject variance (cf. Figure 3e,f). The models with the lowest loss were used for analyses.
**Table S1:** Correlations of Control Energy with Negative Affect (a) and Rumination (b) Scores During Transition to Resting State within the rMDD group.
**Table S2:** Hyperparameters used for DSR model training.
**Table S3:** Medication sensitivity analysis. Descriptive effect sizes for the main rMDD–HC comparisons in the full sample and after excluding medicated rMDD participants. Effect sizes are reported as rank‐biserial correlations (rrb), computed with rMDD as the first group and HC as the second group. Positive values therefore indicate lower values in rMDD than HC, whereas negative values indicate greater values in rMDD than HC.

## Data Availability

All code and fully preprocessed data used for the analyses is openly available at https://github.com/humml‐lab/fmri‐control.
