# Supplementary material for: A Data‐Driven Closed‐Loop Control Approach to Drive Neural State Transitions for Mechanistic Insight
Source: Hum Brain Mapp. 2026 Jul 15;47(11):e70600. doi: 10.1002/hbm.70600 (PMC13370802; doi:10.1002/hbm.70600)
Supplement: Supplementary file 1 — Figure S1: Closed‐loop Control Framework. shPLRNNs inferred on the data model the uncontrolled brain dynamics (center). Control is applied via an additive term ut=uzt (bottom left), optimized to drive the system toward a target state while minimizing control effort (top left). To restrict control to specific brain regions, u~t is projected onto the null space of corresponding rows of the decoder B, using the projection matrix NN⊤ (bottom right). Figure S2: Indication of Nonlinear Separation Between Conditions. (a) To test the contribution of nonlinear information, we trained L2‐regularized logistic regression models on degree‐3 polynomial feature expansions while progressively increasing the number of included terms. Feature sets were built incrementally from linear terms first, then by adding quadratic and cubic interaction terms. Mean test accuracy and standard deviation across subjects are shown as a function of feature count. Performance increases with the addition of quadratic features, indicating the presence of nonlinear structure, but decreases again when cubic terms are included, consistent with overfitting. (b) The zoomed‐in plot illustrates that linear features are not sufficient to obtain accuracies that differ from chance level. Figure S3: (a) UMAP dimensionality reduction applied to states sampled from the Gaussian Mixture Models (GMMs) confirms that the empirical separation is well preserved (cf. Figure 2b). (b) Model selection using AIC and BIC across subjects suggests optimal cluster number of 6. (c) Group comparisons of the distance between GMMs fitted to the resting and sad mood induction states. No significant differences were observed across metrics: L2 Distance (p = 0.81), Maximum Mean Discrepancy (MMD, p = 0.92), and Sliced Wasserstein Distance (SW, p = 0.74). Figure S4: Robustness to Variations in Control Energy Regularization λE and Time Horizon D for Control toward Sad Mood. (a) Without an explicit penalty on control energy (λE = 0), overal [file HBM-47-e70600-s001.pdf]

Supporting Information for
A Data-Driven Closed-Loop Control Approach to Drive Neural State Transitions for Mechanistic Insight

fMRI Preprocessing

Imaging Parameters. Functional imaging was conducted with T2*-weighted EPI scans (TR = 1.5 s, flip angle $\alpha = 80°$, TE = 28 ms, 24 slices, voxel size $3 \times 3 \times 4$ mm^3, 180 volumes per phase), while high-resolution anatomical images were captured using T1-weighted MPRAGE sequences (TR = 2.3 s, TE = 3.03 ms, voxel size $1 \times 1 \times 1$ mm^3). Physiological data, namely pulse and respiration, were recorded at 50 Hz. The first 20 of the 180 images per phase were discarded and preprocessing was conducted with SPM12 (v7738).

Data Preprocessing and Artifact Correction. Preprocessing for the anatomical images included segmentation of the anatomical images and normalization to the ICBM 2009b Nonlinear Asymmetric template, and for the functional images slice timing correction, realignment, coregistration to the anatomical data, normalization to standard space, and spatial smoothing with a FWHM=8x8x8 mm^3 Gaussian kernel.
Onset effects for the key words were modeled by convolving their onset stick function with the canonical hemodynamic response function and included in a regression model further containing the six standard motion parameters, their derivatives, the squared values of them, white matter, cerebrospinal fluid, and global signals ((1)) as well as dummy regressors for volumes affected by small movements (framewise displacement > 0.5mm, global intensity change $z > 4$). We removed these confounds via ridge regression, then extracted the residual time series from each target region and applied the preprocessing pipeline described in the main article.

Physiological Confounds. Since elevated connectivity levels were observed in the rMDD group—and such effects can potentially arise from physiological artifacts (e.g., cardiac and respiratory signals, (2, 3)) — we re-estimated all shPLRNN models after removing these artifacts from the data. Using the TAPAS PhysIO toolbox (4), we extracted subject-specific cardiac and respiratory regressors from recorded physiology time series. We then applied ridge regression (ℓ_2 regularization) to model and subtract these nuisance signals, optimizing the regularization weight via cross-validation and assessing fit on a held-out test set. Importantly, the elevated connectivity patterns not only persisted but in some instances became even more pronounced (Figure S9). To remain conservative and retain the original neural signal structure, all subsequent analyses therefore used the models trained on the uncorrected data.

28 Supplementary Figures

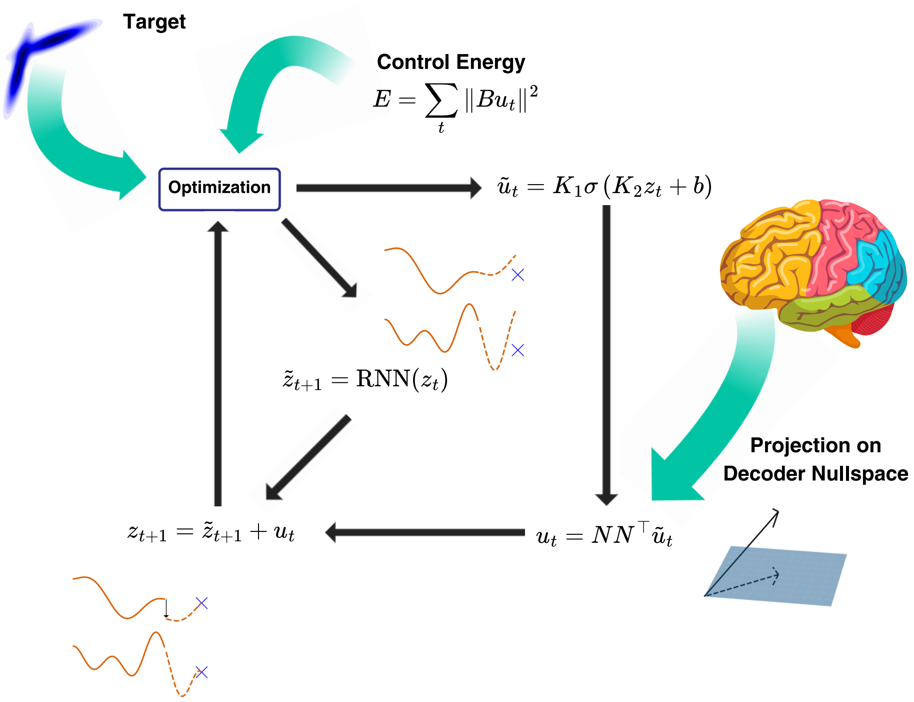

Fig. S1. Closed-loop Control Framework. shPLRNNs inferred on the data model the uncontrolled brain dynamics (center). Control is applied via an additive term $u_t = u(z_t)$ (bottom left), optimized to drive the system toward a target state while minimizing control effort (top left). To restrict control to specific brain regions, $\tilde{u}_t$ is projected onto the null space of corresponding rows of the decoder B, using the projection matrix $NN^\top$ (bottom right).

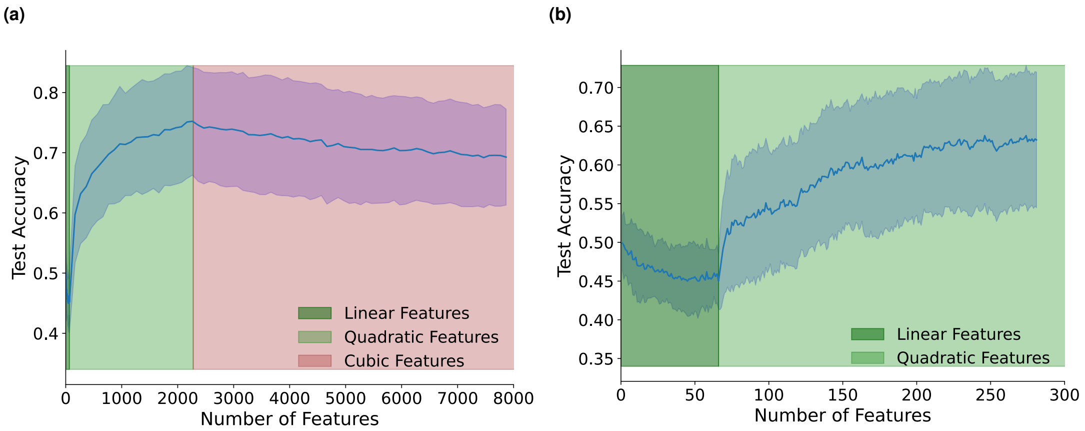

Fig. S2. Indication of Nonlinear Separation Between Conditions. a: To test the contribution of nonlinear information, we trained L^2-regularized logistic regression models on degree-3 polynomial feature expansions while progressively increasing the number of included terms. Feature sets were built incrementally from linear terms first, then by adding quadratic and cubic interaction terms, and model hyperparameter was selected by 5-fold cross-validation before evaluating test accuracy. Mean test accuracy and standard deviation across subjects are shown as a function of feature count. Performance increases with the addition of quadratic features, indicating the presence of nonlinear structure, but decreases again when cubic terms are included, consistent with overfitting. **b:** The zoomed-in plot illustrates that linear features are not sufficient to obtain accuracies that differ from chance level.

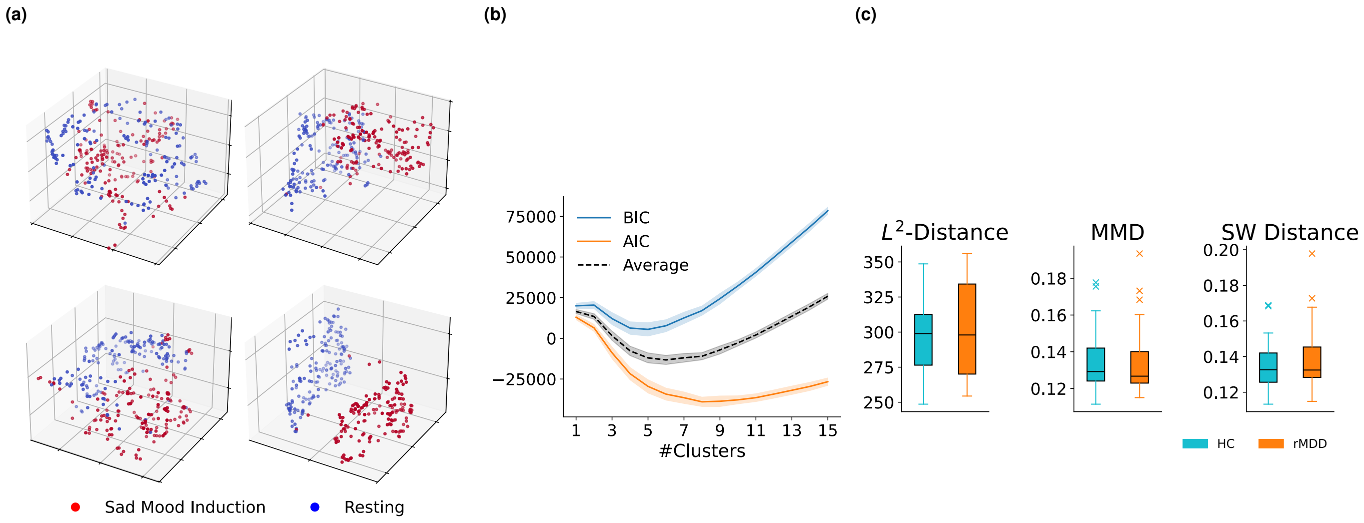

Fig. S3. a: UMAP dimensionality reduction applied to states sampled from the Gaussian Mixture Models (GMMs) confirm that the empirical separation is well preserved (cf. Figure 2b). **b:** Model selection using AIC and BIC across subjects suggests optimal cluster number of 6. **c:** Group comparisons of the distance between GMMs fitted to the resting and sad mood induction states. No significant differences were observed across metrics: L^2 Distance ($p = 0.81$), Maximum Mean Discrepancy (MMD, $p = 0.92$), and Sliced Wasserstein Distance (SW, $p = 0.74$).

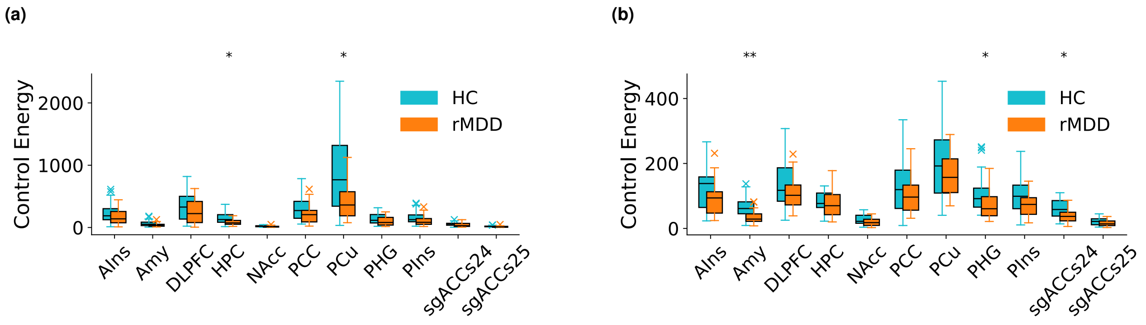

Fig. S4. Robustness to Variations in Control Energy Regularization λ_{E} and Time Horizon D for Control toward Sad Mood. a: Without an explicit penalty on control energy ($\lambda_{\mathrm{E}} = 0$), overall energy levels rise, and the rMDD group generally requires less control (statistically significant within HPC and precuneus; PCu). **b**: With a shorter horizon ($D = 5$), the model has fewer time steps before penalties on control energy and target distance apply; rMDD likewise requires less control energy. **a, b** $^{*}p_{\mathrm{FDR}} < 0.05$.

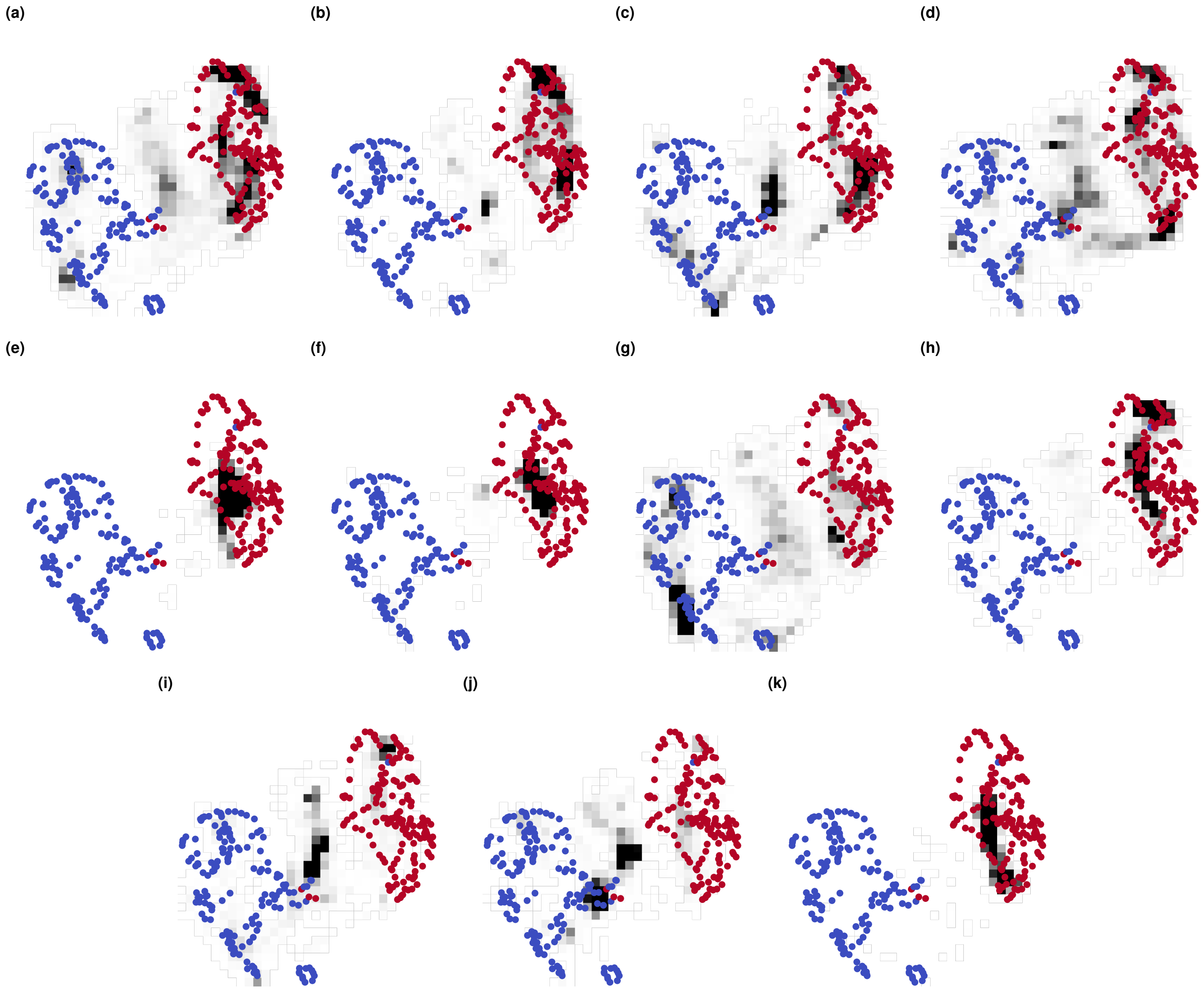

Fig. S5. a-k: Two-dimensional UMAP visualizations of empirical states (sad mood induction in red, resting in blue) and trajectories (black) controlled towards sad mood for different targeted brain regions in a fixed subject. The control successfully maintains activity within the sad mood induction state-space region. Trajectories from multiple initializations are aggregated by spatial binning, and relative frequencies per bin are displayed as a heatmap. Time steps smaller than $D = 10$ were discarded.

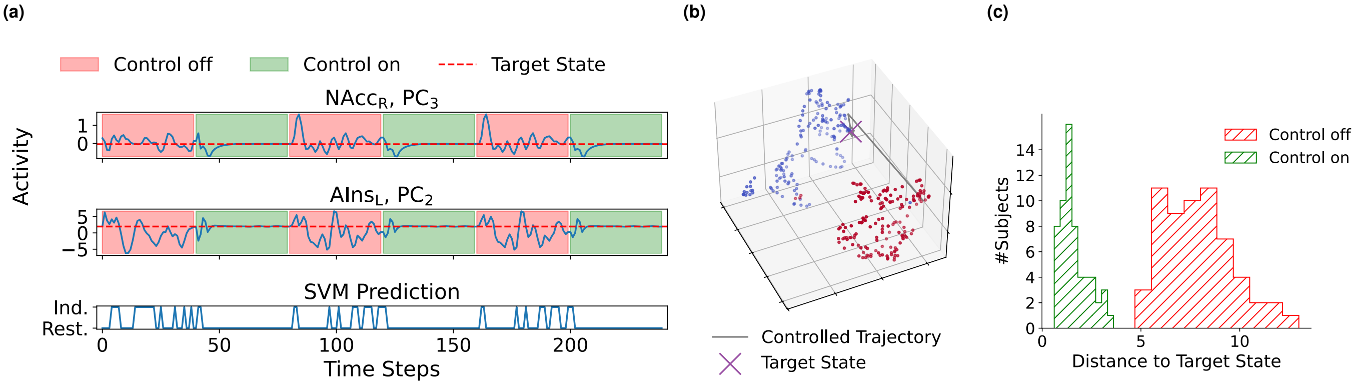

Fig. S6. Hard Target Control: A specific sad mood state (red dashed line) was designated as the target, and control was optimized using mean squared error (MSE) loss. Control was applied to all cortical regions. **a**: When control is activated, trajectories reliably converge toward the target state (red dashed line); when deactivated, dynamics revert to chaotic behavior. This effect is visible in both controlled (right AIns) and uncontrolled (left NAcc) dimensions. The SVM decoder confirms convergence into the resting state region. **b**: UMAP visualization of a trajectory initialized at sad mood induction (red) converging to the target resting state (blue). **c:** Control significantly reduces the Euclidean distance to the target state (median across time), consistently across all subjects.

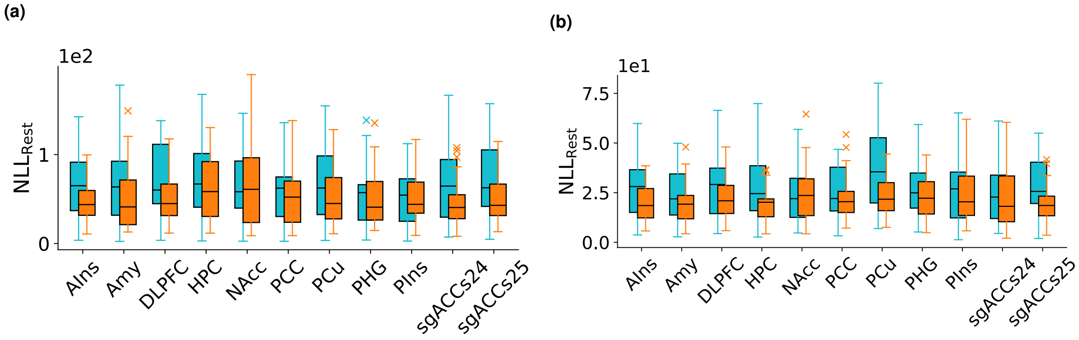

Fig. S7. No Group Differences in Terms of Proximity to Resting States: Both for the transition from *resting state to sad mood* (**a**) and *sad mood to resting state* (**b**), rMDD and HC maintain a similar proximity to the resting state.

(a)

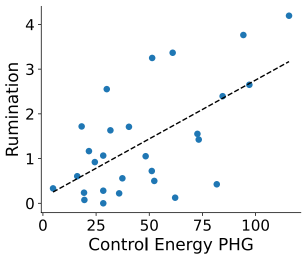

Fig. S8. Correlation Between Rumination Ratings and Control Energy. In rMDD patients, higher rumination ratings are associated with increased control energy required to drive neural activity from sad mood states toward resting states. Shown is control applied to the parahippocampal gyrus (PHG) ($r = 0.62,\ p_{\mathrm{FDR}} < 0.01$).

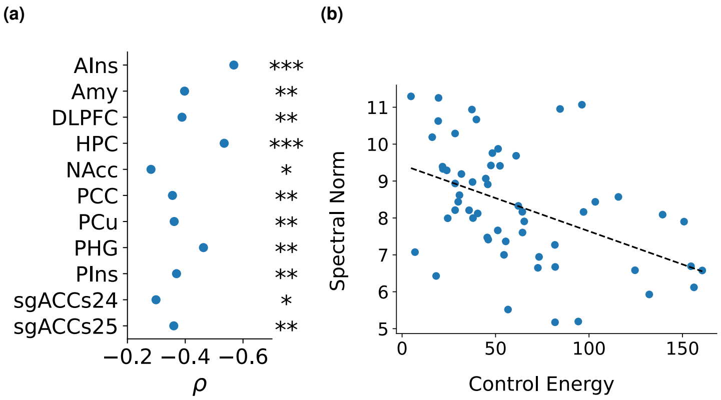

Fig. S9. Correlation for Transitions Toward Resting State. a: For the control in the resting direction, the negative correlation between region coupling and control energy again shows that increased coupling strength is associated with regions with reduced energy expenditure. **b**: Specific values of the spectral norm and the energy for each subject on the example of the PHG (c.f. Figure 5c and 5d).

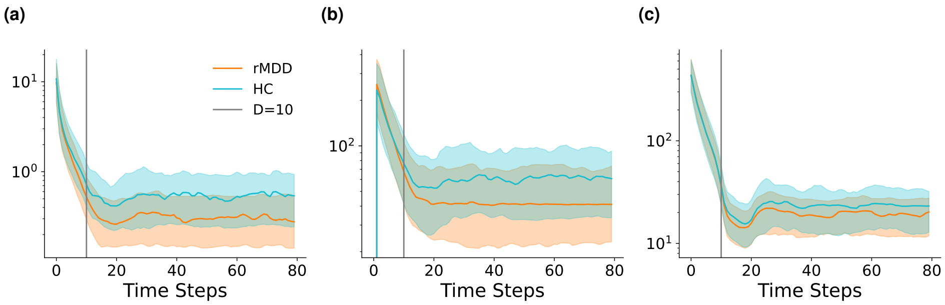

Fig. S10. Temporal Evolution of the Evaluation Measures. Control energy (**a**), $\mathrm{NLL}_{\mathrm{Rest}}$ (**b**), and $\mathrm{NLL}_{\mathrm{Ind}}$ (**c**) for the case of amygdala-targeted control. Curves show the median across subjects and initializations; shaded areas denote the inter-quartile range. While rMDD subjects consistently require less control energy, no significant differences are observed in the likelihoods that quantify the proximity to initial and target states.

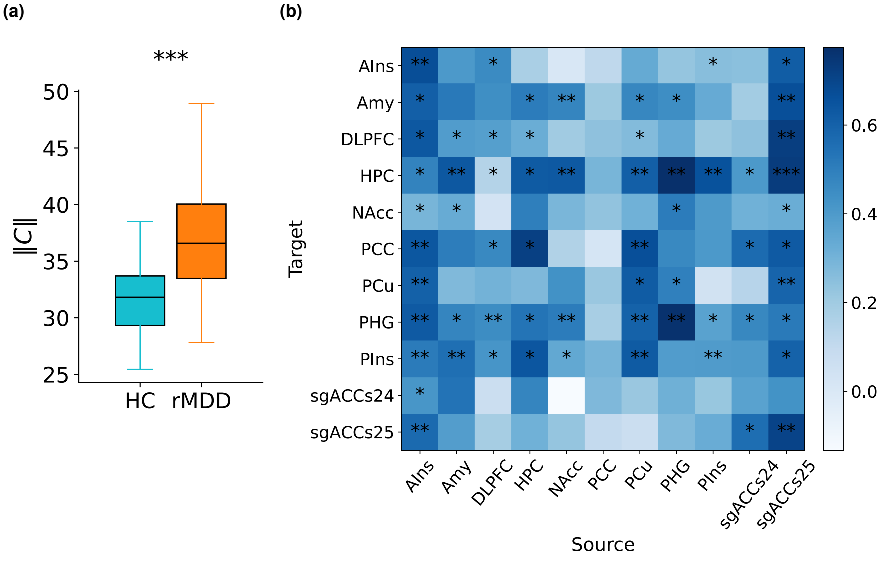

Fig. S11. Connectivity Differences When Training was Performed on Data Corrected for Potential Physiological Artifacts. When correcting the data for physiological effects (i.e. cardiac and respiratory noise) before training the shPLRNNs, the significant difference in connectivity between the groups remains evident. Both the global coupling (**a**) and the pairwise connectivity between brain regions (**b**, difference in median, rMDD-HC) is significantly stronger in rMDD. Here, $^{*}p_{\text{FDR}} < 0.05, ^{**}p_{\text{FDR}} < 0.01, ^{***}p_{\text{FDR}} < 0.001$.

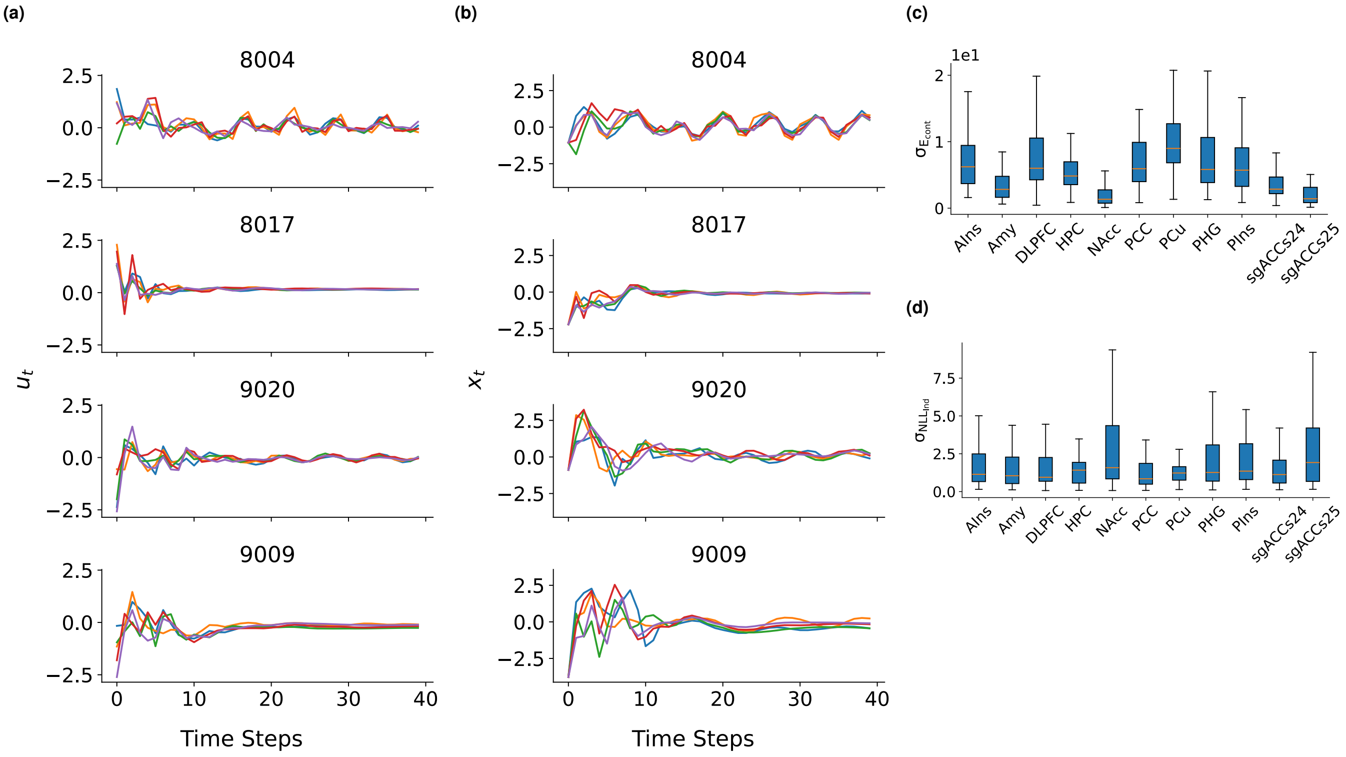

Fig. S12. Robustness of Control Parameters to Initialization. For each experiment and subject, five models were independently optimized. Controlled trajectories show predominantly consistent control inputs u_t (**a**) and corresponding activity x_t (**b**) across runs, each represented by a separate color-coded curve, displayed for a selected number of subjects. While the chaotic dynamics amplify small deviations over time, variance across runs of control energy (**c**) and $\mathrm{NLL}_{\mathrm{Ind}}$ (d) remains substantially smaller than inter-subject variance (cf. Figure 3e, 3f). The models with the lowest loss were used for analyses.

Table S1. Correlations Between Control Energy with Negative Affect (a) and Rumination (b) Scores During Transition to Resting State within the rMDD group.

Region	r	p_{FDR}
AIns	0.30	0.157
Amy	0.39	0.067
DLPFC	0.35	0.088
HPC	0.53	0.009
NAcc	0.59	0.006
PCC	0.17	0.355
PCu	0.22	0.278
PHG	0.45	0.038
PIns	0.44	0.038
sgACCs24	0.36	0.088
sgACCs25	0.58	0.008

(a) Negative Affect

Region	r	p_{FDR}
AIns	0.03	0.871
Amy	0.38	0.109
DLPFC	0.34	0.135
HPC	0.17	0.505
NAcc	0.22	0.383
PCC	0.08	0.734
PCu	0.11	0.685
PHG	0.62	0.003
PIns	0.52	0.013
sgACCs24	0.60	0.003
sgACCs25	0.37	0.126

(b) Rumination

Table S2. Hyperparameters used for DSR model training.

M	L	α_0	α_1	β_1	β_2	λ_1	λ_2	λ_3	λ_4	η_0	η_1
66	15	10^{-1}	10^{-3}	0.9	0.999	10^{-1}	0	10^{-4}	10^{-4}	10^{-3}	10^{-4}

Table S3. Medication sensitivity analysis. Descriptive effect sizes for the main rMDD–HC comparisons in the full sample and after excluding medicated rMDD participants. Effect sizes are reported as rank-biserial correlations (r_{rb}), computed with rMDD as the first group and HC as the second group. Positive values therefore indicate lower values in rMDD than HC, whereas negative values indicate greater values in rMDD than HC.

Measure		Full sample	Unmedicated	Full sample	Unmedicated
Coupling	Global	-0.52	-0.56		
		rest→ sad	rest→ sad	sad→ rest	sad→ rest
Energy	AIns	0.36	0.34	0.54	0.36
	Amy	0.36	0.32	0.36	0.32
	DLPFC	0.40	0.41	0.29	0.26
	HPC	0.49	0.48	0.38	0.37
	NACC	0.03	0.08	0.28	0.14
	PCC	0.29	0.29	0.09	0.08
	PCU	0.45	0.40	0.20	0.18
	PHG	0.41	0.36	0.40	0.25
	PIns	0.51	0.61	0.25	0.15
	sgACCs24	0.51	0.48	0.32	0.23
	sgACCs25	0.44	0.36	0.27	0.18
$\mathrm{NLL}_{\mathrm{Ind}}$	AIns	0.15	0.10	0.49	0.45
	Amy	0.18	0.16	0.40	0.39
	DLPFC	0.39	0.25	0.30	0.19
	HPC	0.30	0.29	0.28	0.29
	NACC	0.15	0.40	0.47	0.39
	PCC	0.10	0.11	0.35	0.33
	PCU	0.27	0.34	0.39	0.34
	PHG	0.24	0.24	0.35	0.31
	PIns	0.25	0.38	0.21	0.19
	sgACCs24	0.27	0.22	0.28	0.37
	sgACCs25	0.32	0.27	0.31	0.22
$\mathrm{NLL}_{\mathrm{Rest}}$	AIns	0.32	0.35	0.31	0.20
	Amy	0.22	0.18	0.16	0.20
	DLPFC	0.24	0.19	0.22	0.01
	HPC	0.07	0.07	0.33	0.32
	NACC	-0.01	0.05	-0.03	-0.17
	PCC	0.10	0.12	0.10	0.10
	PCU	0.17	0.18	0.38	0.36
	PHG	0.09	0.01	0.16	0.10
	PIns	0.04	0.07	0.05	-0.07
	sgACCs24	0.34	0.31	0.14	0.03
	sgACCs25	0.25	0.14	0.37	0.18

References

1. L Parkes, B Fulcher, M Yücel, A Fornito, An evaluation of the efficacy, reliability, and sensitivity of motion correction strategies for resting-state functional MRI. *NeuroImage* **171**, 415–436 (2018).
2. M Wilding, A Ischebeck, N Zaretskaya, Respiration recording for fMRI: breathing belt versus spine coil sensor. *Imaging Neurosci.* **2**, 1–11 (2024).
3. VE Zamoscik, et al., Respiration pattern variability and related default mode network connectivity are altered in remitted depression. *Psychol. Medicine* **48**, 2364–2374 (2018).
4. L Kasper, et al., The PhysIO Toolbox for Modeling Physiological Noise in fMRI Data. *J. Neurosci. Methods* **276**, 56–72 (2017).
